# Myeloperoxidase impacts vascular function by altering perivascular adipocytes’ secretome and phenotype in obesity

**DOI:** 10.1016/j.xcrm.2025.102087

**Published:** 2025-04-18

**Authors:** Alexander Hof, Max Landerer, Philipp Peitsmeyer, Ronja Herzog, Jens Alber, Maysam Ahdab, Felix Sebastian Nettersheim, Dennis Mehrkens, Simon Geißen, Simon Braumann, Henning Guthoff, Philipp von Stein, Harshal Nemade, Felix Simon Ruben Picard, Ramona Braun, Friedrich Felix Hoyer, Jens Claus Brüning, Alexander Pfeifer, Staffan Hildebrand, Holger Winkels, Stephan Baldus, Matti Adam, Jasper Schäkel, Martin Mollenhauer

**Affiliations:** 1 Heart Center, Department of Cardiology, Faculty of Medicine and University Hospital Cologne, University of Cologne, 50937 Cologne, Germany; 2 Department of Cardiology, University Heart and Vascular Center Hamburg, 2024 Hamburg, Germany; 3 La Jolla Institute for Immunology, La Jolla, CA 92037, USA; 4 Institute of Pharmacology and Toxicology, University Hospital Bonn, University of Bonn, 53127 Bonn, Germany; 5 Center for Molecular Medicine Cologne (CMMC), University of Cologne, 50937 Cologne, Germany; 6 Max Planck Institute for Metabolism Research, 50937 Cologne, Germany

**Keywords:** obesity, myeloperoxidase, perivascular adipose tissue, inflammation, adiponectin, endothelial function

## Abstract

Obesity, a main driver of cardiovascular morbidity, contributes to endothelial dysfunction and inflammation in adipose tissues. Perivascular adipose tissue (PVAT) surrounds arteries and influences vascular function. In obesity, immune cells, including myeloperoxidase (MPO)-releasing myeloid cells, accumulate in PVAT. In this study, we show MPO levels to correlate with body weight and endothelial function in obese patients (*n* = 33) and mice. In addition, MPO deficiency reduces immune cell frequency, enhances PVAT beiging via soluble guanylyl cyclase β1 (sGC-β1), and increases oxygen consumption *in vivo*. Further, nitrotyrosine formation and inflammatory cytokine release are attenuated in obese *Mpo*^−/−^ mice. Mechanistically, adiponectin (APN) secretion improves endothelial function and reduces arterial stiffness. *In vitro*, MPO-treated human white adipocytes show lower APN and brown adipocyte marker expression but increased inflammation. Thus, MPO impairs vascular function via PVAT inflammation and suppression of vasoprotective mediators, making it a potential therapeutic target in obesity-related cardiovascular disease.

## Highlights

• MPO is associated with endothelial dysfunction in obese patients
• MPO induces pro-inflammatory PVAT activation and immune cell abundance
• MPO suppresses PVAT beiging via sGC-β1 inhibition, thereby reducing oxygen consumption
• MPO is a potential therapeutic target to prevent cardiovascular disease in obesity

## Graphical abstract

**Figure:**
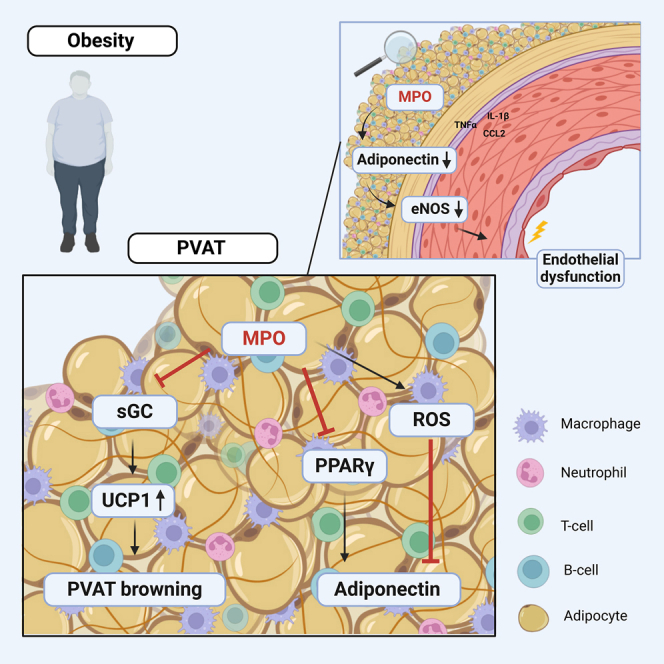

## Teaser

Hof et al. show that myeloperoxidase (MPO) links obesity to vascular dysfunction by driving perivascular adipose tissue (PVAT) inflammation. MPO levels correlate with body weight and endothelial dysfunction, while MPO deficiency reduces PVAT inflammation, promotes adipocyte “beiging” via sGC-β1, increases adiponectin secretion, and improves vascular function.

## Introduction

Obesity is a globally and rapidly expanding epidemic and represents a major risk factor for numerous pathologies such as atherosclerosis, coronary artery disease, atrial fibrillation, insulin resistance, and stroke.^1^^,^^2^ In 2015, obesity contributed to 4.0 million deaths of which two-thirds were attributable to cardiovascular diseases (CVDs).^3^ Hence, treatment, prevention, and comprehending underlying pathomechanisms of obesity-induced CVD are of highest therapeutic and socioeconomic importance. Obesity promotes overall metabolic inflammation, specifically in adipose tissues. Several studies have shown endothelial dysfunction, a dysregulated vascular tone, and an imbalanced endothelial redox and inflammatory state in obesity.^4^^,^^5^ Endothelial dysfunction represents an early stage of vascular damage, from which atherosclerosis and major adverse cardiovascular events may arise.^6^^,^^7^

Different types of adipose tissue exist in various locations with diverse ontogenetic origins and metabolic and endocrine properties in mammalians. White adipose tissue (WAT) makes up the largest part of body fat mass in human adults and expands in obesity. It is responsible for energy storage and hormone and adipokine production and secretion.^8^ Brown adipose tissue (BAT) is characterized by its ability to generate heat by energy dissipation. It plays a predominant role in protecting newborns from hypothermia by non-shivering thermogenesis via the mitochondrial uncoupling protein 1 (UCP-1), but BAT depots regress gradually during childhood.^8^ Some WATs can adapt characteristics of BAT, for example, expression of UCP-1 and non-shivering thermogenesis, in a process called “beiging.” One of the tissues with high beiging capacity in adults is perivascular adipose tissue (PVAT), which is transcriptionally similar to BAT.^9^^,^^10^ First considered exclusively as structurally supportive tissue surrounding the vessel wall, recent findings identified PVAT as a modulator of vascular tone and function. PVAT is an active endocrine and paracrine organ secreting adipokines and other vasoactive substances like nitric oxide (NO) and cytokines.^11^ In steady state, vasoprotective mediators such as adiponectin (APN) profoundly contribute to an intact vascular function, while obesity provokes disequilibrium in PVAT secretome, favoring the release of vasodegenerative compounds.^11^^,^^12^

Adipose tissues host a variety of immune cells. In obesity, a pro-inflammatory state of fat depots is induced, characterized by elevated immune cell infiltration and secretion of pro-inflammatory cytokines like tumor necrosis factor (TNF)-α, interleukin (IL)-1, and IL-6.^13^ Myeloperoxidase (MPO), a bactericidal and fungicidal heme-enzyme, is mainly produced by polymorphonuclear neutrophils (PMNs) and released upon cell activation. MPO catalyzes the production of highly reactive hypochlorous acid (HOCl) by oxidation of its substrate H_2_O_2_.^14^ Yet, MPO has detrimental effects on the cardiovascular system, fosters the development of atherosclerosis, and is a valid risk predictor in patients with acute coronary syndrome and other CVDs.^15^^,^^16^ MPO exerts various effects on the luminal side of the vessel wall. Underlying mechanisms are increased cellular oxidative stress, reduction in NO bioavailability, immune cell recruitment, and disruption of glycocalyx integrity.^14^^,^^16^^,^^17^

Studies that investigate abluminal effects of MPO on the vessel wall, and particularly on the function of PVAT as an imbalanced endocrine organ in obesity, are lacking. Thus, revelation of MPO’s contribution to endothelial dysfunction “from the outside to the inside” of the vessel wall forms the scope of this work.

## Results

### Decline in MPO plasma levels after bariatric surgery improves endothelial function

**Figure 1:**
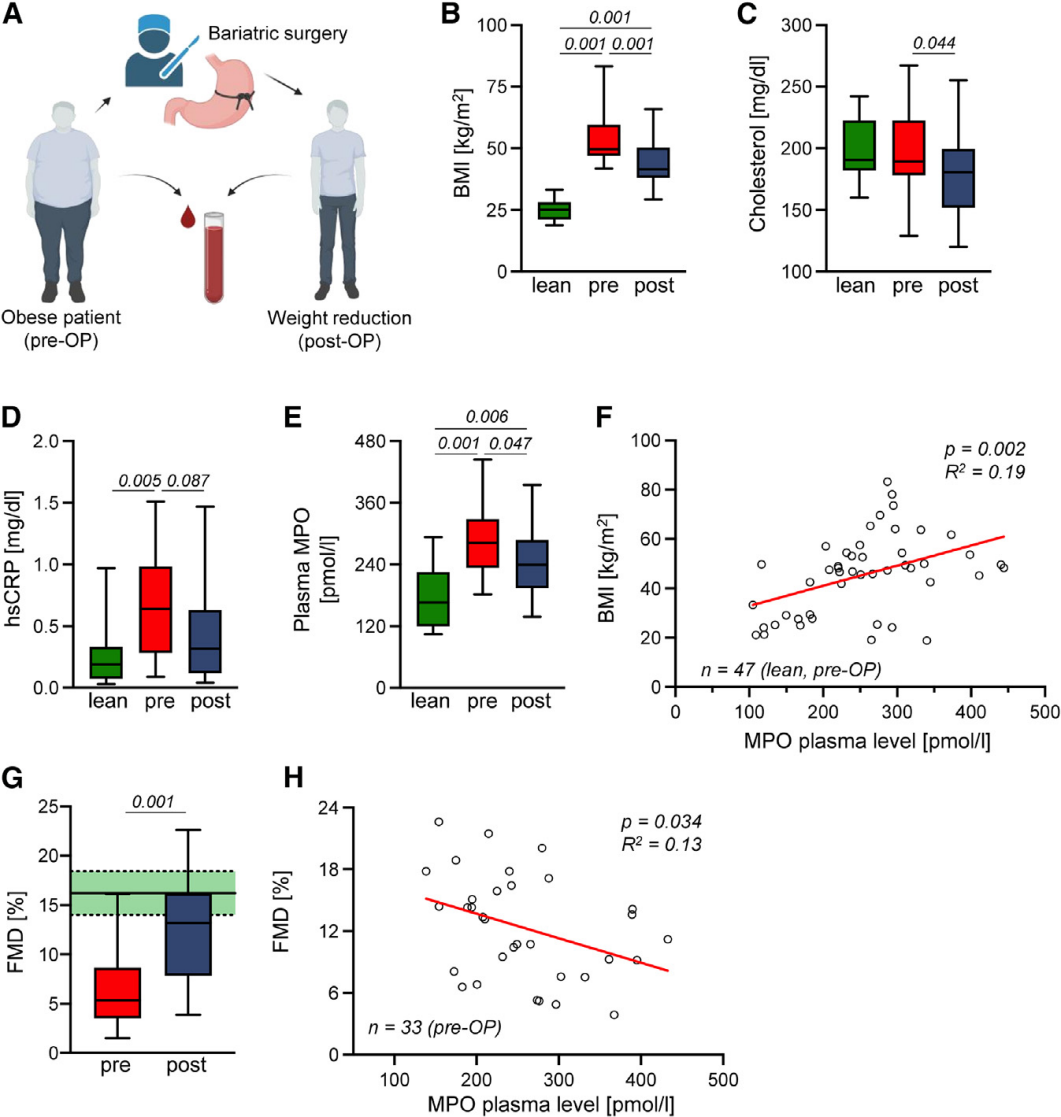
Bodyweight loss reduces inflammatory state and improves endothelial function in bariatric patients Schematic overview of the experimental setup (A). Obese patients with a BMI > 37 kg/m^2^ underwent bariatric surgery by Roux-en-Y gastric bypass or gastric banding. Blood samples were obtained, and flow-mediated dilation (FMD) was measured before (pre) and 3 months after (post) bariatric surgery. Lean patients were included for blood analyses as non-obese controls. BMI (B), blood cholesterol levels (C), and plasma high-sensitive CRP (hsCRP) (D) before and after bariatric surgery and in lean control patients. MPO plasma levels (E) and correlation of BMI and MPO plasma levels (F). FMD analysis before and after bariatric surgery (G, green area indicates FMD standard value range^18^) and correlation of FMD and MPO plasma levels (H). *n* (patients) = 33. *n* (lean controls) = 14. For (E) and (G), pre- and post-OP values were analyzed. *p* as indicated. Statistical significance was determined by ordinary one-way ANOVA followed by Tukey’s multiple comparison test (C, E, and G) and Kruskal-Wallis followed by Dunn’s multiple comparison test (B and D) or simple linear regression using a Pearson’s correlation calculation (F and H).

50 obese patients with a BMI >37 kg/m^2^ were initially included and underwent bariatric surgery via gastric bypass or sleeve gastrectomy (Figure 1A). Of those, 17 patients were lost to follow-up or retracted study consent. 14 lean patients served as control cohort. Overall patient characteristics are presented in Table S1. The body mass index (BMI) was significantly reduced by 18.24% with a median body weight reduction of 9 kg/m^2^ 3 months after surgery (Figure 1B). Likewise, total cholesterol levels decreased significantly (Figure 1C), mainly driven by a decrease in triglyceride levels between pre- and post-operative (OP) plasma levels (Figures S1A–S1C). To assess the inflammatory status, plasma high-sensitive C-reactive protein (hsCRP) levels were determined, which were significantly higher in obese patients than in lean controls but numerically decreased after bariatric surgery (Figure 1D). Furthermore, total MPO plasma levels were increased in obese individuals compared to lean controls but significantly declined after surgery (Figure 1E). MPO levels (lean and pre-OP) correlated positively with BMI (Figure 1F).

Subsequently, we analyzed whether bariatric surgery in patients with obesity affects endothelial function by measuring flow-mediated vasodilatation (FMD). Weight reduction led to a significantly improved endothelial function, increasing mean FMD from 6.7% to 11.7%, almost reaching FMD values of a young, healthy control cohort previously published^18^ (Figure 1G). Of note, MPO plasma levels correlated negatively with FMD in bariatric patients (Figure 1H). MPO levels did not differ significantly with respect to smoker status, diabetes, or age in the obese patient cohort (Figures S1D–S1F). Single data point graphs are shown in Figure S2.

### MPO promotes vascular dysfunction in obese mice

**Figure 2:**
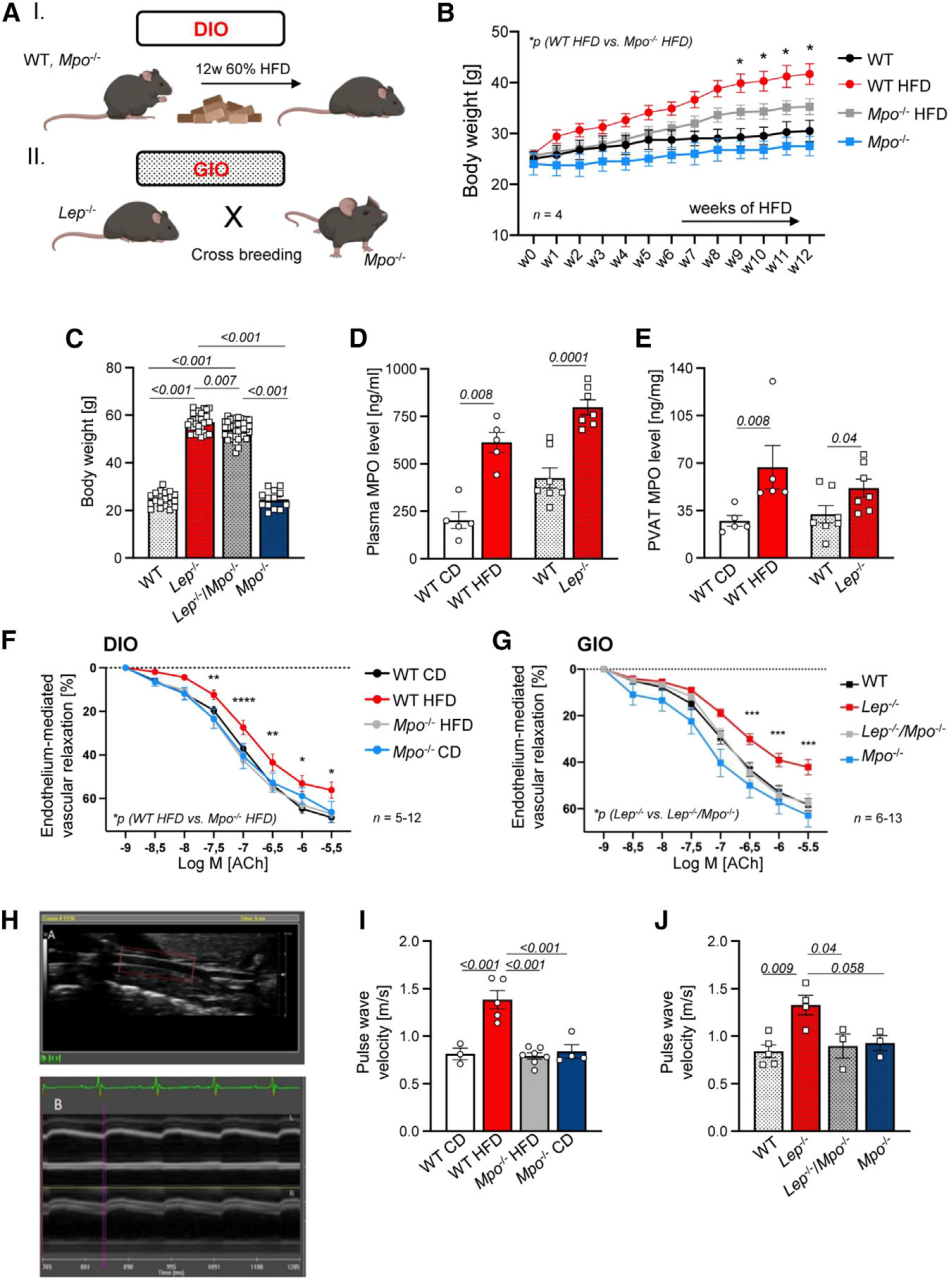
MPO impacts vascular function in murine models of obesity Schematic overview of the experimental setup (A). For dietary-induced obesity (DIO), wild-type (WT) and *Mpo*^−/−^ mice were fed a 60% HFD for 12 weeks. Lepti*n* (*Lep*)^−/−^ mice with genetically inherited obesity (GIO) were crossbred with *Mpo*^−/−^ mice. Lean WT and *Mpo*^−/−^ littermates of the respective line were used as controls. Body weight curve of HFD-fed WT and *Mpo*^−/−^ mice and lean controls over 12 weeks starting at 8 weeks of age; *n* = 4 (B). Body weight of WT, *Lep*^−/−^, *Lep*^−/−^/*Mpo*^−/−^, and *Mpo*^−/−^ mice at 14 weeks of age (C). MPO levels in plasma (D) and in perivascular adipose tissue (PVAT) (E) of obese mice and lean controls in the DIO (left) and GIO (right) model. Acetylcholine-depended endothelium-mediated vascular relaxation of isolated aortic rings of DIO (F) and GIO mice (G) and lean controls measured by organ bath investigation. Representative images of vascular ultrasound of carotid arteries (H). Pulse wave velocity (PWV) analyses in obese mice and lean controls in the DIO (I) or GIO (J) model. Data are presented as mean ± SEM. *n* = as indicated. Statistical significance was determined by unpaired Student’s t test (D and E) or by ordinary two-way ANOVA followed by Tukey’s multiple comparison test (B, C, F, G, I, and J). (B, F, and G) *p =* WT HFD vs. *Mpo*^−/−^*HFD or Lep*^−/−^ vs. *Lep*^−/−^/*Mpo*^−/−^, respectively. ∗*p* < 0.05; ∗∗*p* < 0.01; ∗∗∗*p* < 0.001; ∗∗∗∗*p* < 0.0001. *n* = 5–13

A dietary-induced obesity (DIO) and genetically inherited obesity (GIO) mouse model were implemented to study the involvement of MPO in obesity-related endothelial dysfunction (Figure 2A). Weight gain was significantly higher in MPO-competent wild-type (WT) mice compared to *Mpo*^−/−^ littermates fed a high-fat diet (HFD) for 12 weeks (Figures 2B and S3). Accordingly, 14 week-old *Lep*^−/−^ mice were heavier in comparison to *Lep*^−/−^/*Mpo*^−/−^ mice (Figure 2C). Murine PVAT and thoracic aortic tissue were isolated (Figure S4). DIO and GIO mice showed 3-fold elevated MPO plasma levels and higher MPO concentrations in PVAT as compared to control diet (CD)-fed or WT littermates, respectively (Figures 2D and 2E). In the aortic wall, heightened MPO levels were only observed in DIO mice (Figure S5A). In line with endothelial dysfunction observed in patients suffering from obesity, organ bath experiments revealed an impaired endothelium-mediated vascular relaxation in isolated aortic rings of HFD-fed WT mice as well as in obese *Lep*^−/−^ mice, which was not observable in obese *Mpo*^−/−^ mice (Figures 2F and 2G). The dysfunctional vascular response was more severe in *Lep*^−/−^ mice with a vascular relaxation rate of only 40% compared to 55% in DIO mice. To investigate the physiological relevance of these findings *in vivo*, ultrasound analyses of the carotid arteries were performed. Pulse wave velocity (PWV), a surrogate of arterial stiffness, was increased to about 1.5 m/s in both obesity models, whereas MPO deficiency alleviated PWV (Figures 2H–2J). In addition, intima media thickness (IMT) was increased in *Lep*^−/−^ but not in *Lep*^−/−^/*Mpo*^−/−^ mice (Figure S5B).

In a translational study, treatment with the pharmacological MPO inhibitor 4-amino-benzhydrazide (4-ABAH, MPOi) or a vehicle control every other day for 2 weeks showed no effect of MPOi on body weight gain (Figures S6A and S6B). Organ bath investigations revealed no differences between MPOi- and vehicle-treated aortas (Figure S6C). Nonetheless, sonographic investigations of the carotid artery showed an improved PWV and a numerically reduced IMT in this translational approach when obese WT mice were treated with MPOi (Figures S6D and S6E).

### Myeloid cell recruitment into PVAT is attenuated in obese MPO-deficient mice

**Figure 3:**
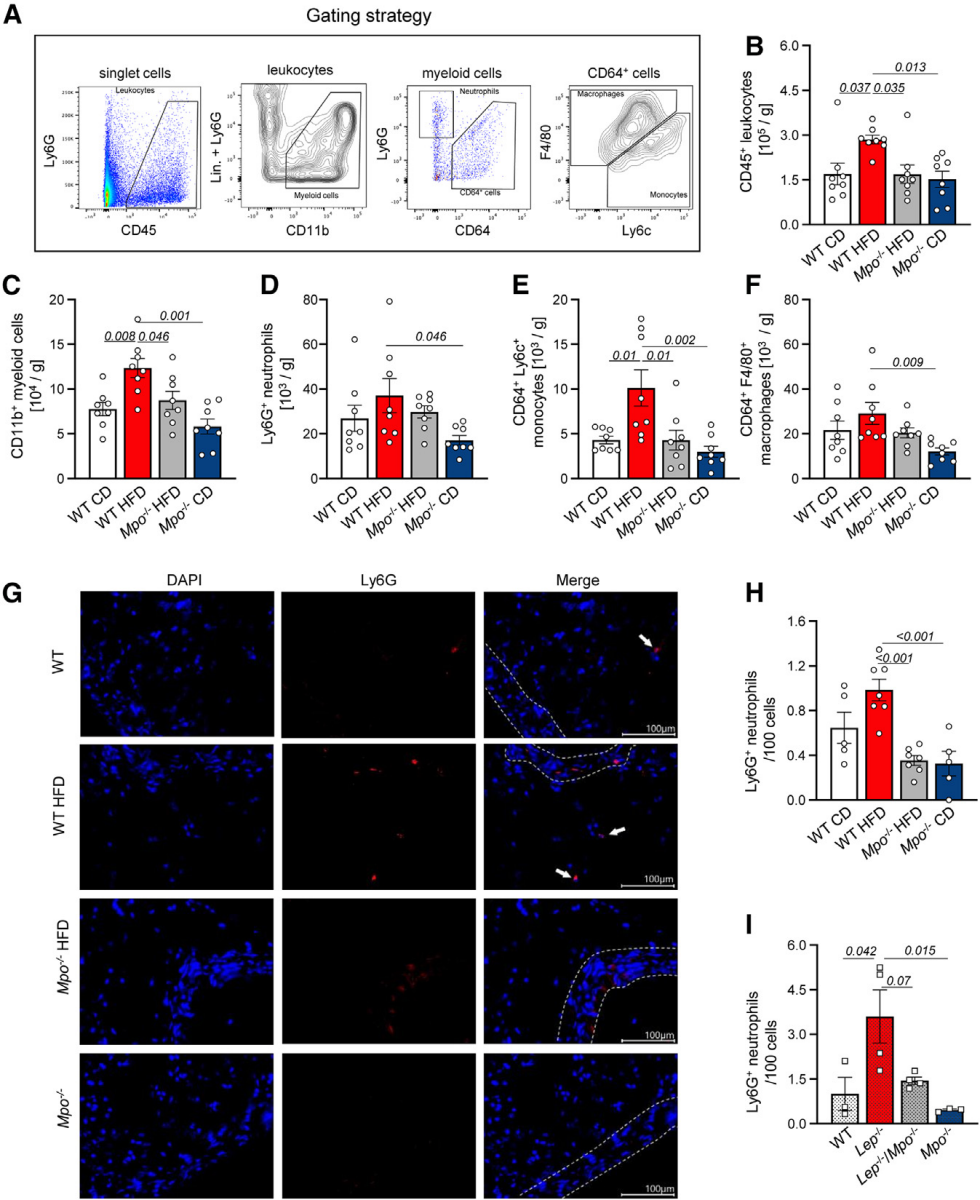
MPO deficiency reduces myeloid cell frequency in perivascular adipose tissue after high-fat diet Leukocyte populations in DIO PVAT were analyzed by flow cytometry (A: gating strategy). Counts of CD45^+^ leukocytes (B), CD45^+^CD11b^+^ myeloid cells (C), CD45^+^CD11b^+^CD64^+^Ly6G^+^ neutrophils (D), CD45^+^CD11b^+^CD64^+^Ly6c^+^ monocytes (E), and CD45^+^CD11b^+^CD64^+^F4/80^+^ macrophages (F) are shown. Representative immunohistochemical stainings of Ly6G^+^ neutrophils in PVAT of DIO mice (G). White arrows indicate Ly6G^+^ neutrophils. Ly6G count in the PVAT of DIO (H) and GIO (I) mice. *n* = as indicated. Data are presented as mean ± SEM. Statistical significance was determined by ordinary two-way ANOVA followed by Tukey’s multiple comparison test.

In obesity, immune cells accumulate in adipose tissues. We thus analyzed leukocyte and myeloid cell populations in PVAT of obese GIO and DIO mice by flow cytometry. CD45^+^ leukocytes and CD11b^+^ myeloid cell numbers (gating strategy shown in Figure 3A) were significantly reduced in PVAT of obese *Mpo*^−/−^ mice as compared to obese WT littermates (Figures 3B and 3C). Further subpopulation analysis revealed a decrease of CD64^+^ Ly6C^+^ monocytes in the PVAT of obese *Mpo*^−/−^ mice as compared to obese WT animals (Figures 3D–3F). Similar effects were observed within the PVAT of GIO mice, showing significantly increased numbers of CD45^+^ leukocytes, CD11b^+^ myeloid cells, Ly6G^+^ neutrophils, Ly6c^+^ monocytes, and CD64^+^ F4/80^+^ macrophages in *Lep*^−/−^ mice, but not in *Lep*^−/−^*/Mpo*^−/−^ mice (Figure S7). We observed significant neutrophil infiltration into PVAT of obese mice histologically. MPO deficiency reduced PVAT neutrophil counts in both models to control level (DIO: Figures 3G and 3H; GIO: Figures 3I and S8). The number of CD68^+^ macrophages followed a similar trend in PVAT of WT, but not in *Mpo*^−/−^ mice fed an HFD (Figures S9A and S9B).

Blood immune cell analysis by flow cytometry revealed elevated numbers of CD115^+^ Ly6C^+^ monocytes in *Lep*^−/−^ mice compared to *Lep*^−/−^*Mpo*^−/−^ mice and lean controls, whereas Cd11b^+^ cell and Ly6G^+^ neutrophil counts were unaltered (Figures S10A–S10C). Similar results were obtained from DIO mice (Figure S10D) and in part by hematological analysis (Figure S10E).

### MPO mediates inflammation and reactive nitrogen production in obese PVAT

**Figure 4:**
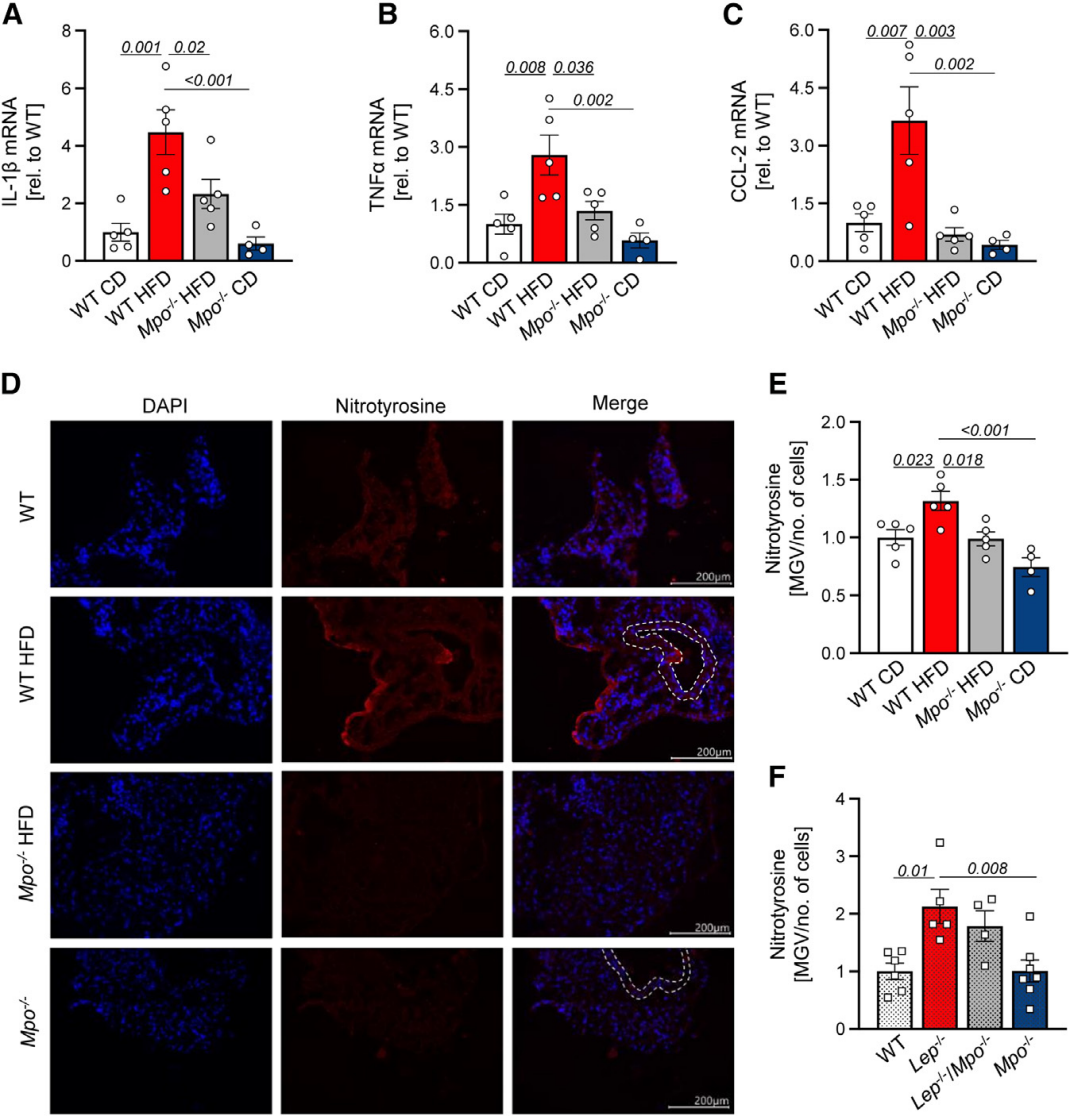
Myeloperoxidase mediates nitrosative tissue damage and pro-inflammatory signaling of PVAT in obesity Gene expression of IL-1β, TNF-α, and CCL-2 in PVAT of DIO mice (A–C). Representative immunohistochemical stainings of nitrosative tissue damage in the PVAT of obese WT and *Mpo*^−/−^ mice and in lean controls (D). Evaluation of nitrotyrosine in the PVAT of DIO (E) and GIO (F) mice. Data are presented as mean ± SEM. Statistical significance was determined by ordinary two-way ANOVA followed by Tukey’s multiple comparison test. *n* = as indicated.

Elevated myeloid cell recruitment in PVAT is a hallmark of inflammation in obesity, which was reduced in MPO-deficient mice. We thus tested whether the expression of chemokines and cytokines was also altered in PVAT. qPCR analyses showed significantly increased mRNA expression of the pro-inflammatory cytokines IL-1β, TNF-α, and CC-chemokine ligand 2 (CCL-2, also known as monocyte chemoattractant protein 1) in PVAT of DIO mice compared to obese *Mpo*^−/−^ mice and chow diet-fed controls (Figures 4A–4C). Interestingly, IL-1β, TNF-α, and CCL-2 mRNA expression was diminished by over 50% in HFD-fed *Mpo*^−/−^ mice as compared to obese WT mice, with expression of the neutrophil-attracting chemokine CCL-2 reaching baseline levels of lean mice. Accordingly, protein levels of pro-inflammatory chemokines were reduced numerically, though not significantly, in the PVAT of HFD-fed *Mpo*^−/−^ mice compared to WT (Figure S11), whereas no major changes could be detected between *Lep*^−/−^ and *Lep*^−/−^*Mpo*^−/−^ PVAT (Figure S12).

To investigate the impact of enhanced MPO-mediated leukocyte abundance in oxidative PVAT modification, we assessed 3-nitrotyrosine as a marker of PMN activity and oxidative stress by immunofluorescence stainings (Figure 4E). 3-nitrotyrosines, generated by reactive nitrogen species via the neutrophilic MPO-H_2_O_2_-NO_2_^−^ system, are not exclusive but characteristic nitrosylation products of MPO.^19^ Of note, 3-nitrotyrosine formation was significantly increased in HFD-fed WT mice compared to *Mpo*^−/−^ mice and lean controls (Figure 4F). Similarly, 3-nitrotyrosine formation was significantly elevated in *Lep*^−/−^ mice, but not in *Lep*^−/−^*Mpo*^−/−^ mice, as compared to lean controls (Figures 4F and S13).

### MPO affects the adipocyte phenotype in obesity

**Figure 5:**
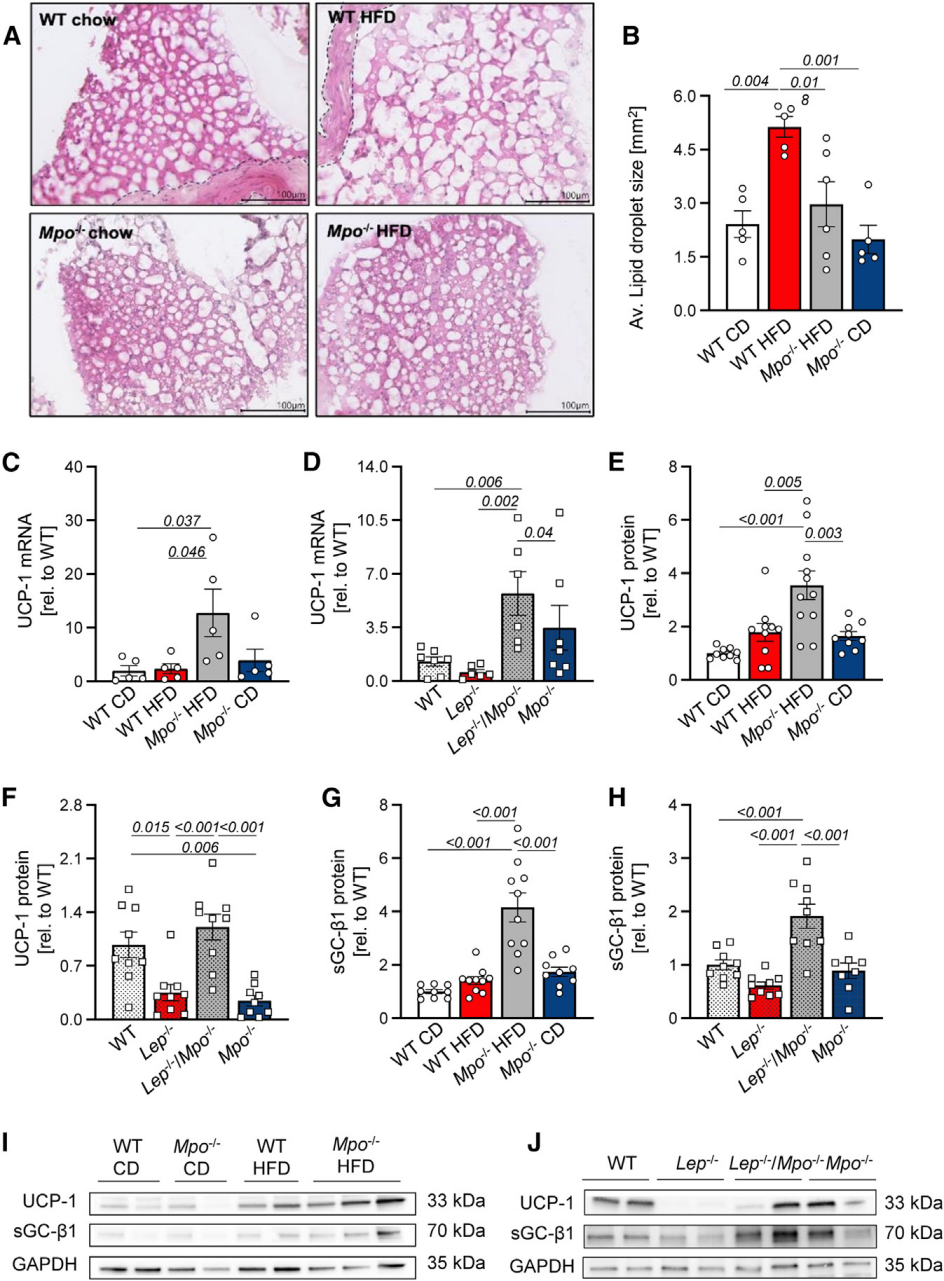
MPO impedes PVAT beiging by inhibition of soluble guanylyl cyclase β1 PVAT morphology of DIO WT and *Mpo*^−/−^ was assessed by automated quantification of lipid vacuoles in hematoxylin and eosin stainings (A: representative H&E stainings and automated binarization; B: results of lipid droplet quantification). UCP-1 mRNA (C and D), UCP-1 protein (E and F), and sGC-β1 protein expression in DIO and GIO PVAT (G and H). Representative immunoblots of UCP-1, sGC-β1, and GAPDH from DIO (I) and GIO (J) PVAT. GAPDH was used as loading control. Data are presented as mean ± SEM. Statistical significance was determined by ordinary two-way ANOVA followed by Tukey’s multiple comparison test. *n* = as indicated. UCP-1, uncoupling protein 1; sGC-β1, soluble guanylyl cyclase β1; GAPDH, glyceraldehyde 3-phosphate dehydrogenase.

Inflammation and metabolic changes associated with obesity alter the phenotype of adipocytes. Hence, we investigated whether MPO is a modulator in this crosstalk. In healthy individuals, PVAT resembles a beige adipocyte phenotype, whereas obesity induces “white shifting” of PVAT accompanied by a metabolic and paracrine disbalance.^11^ We therefore analyzed the morphology of adipocytes in PVAT of obese WT and *Mpo*^−/−^ mice and in lean controls. Automated quantification of lipid droplet size revealed larger lipid vacuoles, a hallmark of WAT, in DIO mice compared to obese *Mpo*^−/−^ mice and lean control animals (Figures 5A and 5B). To further assess the effect of adipocyte phenotypic changes, we analyzed gene and protein expression of the BAT marker UCP-1. UCP-1 was significantly upregulated in PVAT of obese *Mpo*^−/−^ mice compared to obese WT mice in the DIO and GIO model both on transcriptional (Figures 5C and 5D) and on protein level (Figures 5E and 5F). In line with these findings, ASC-1, a WAT marker, was elevated in PVAT of obese *Lep*^−/−^ mice but not in *Lep*^−/−^/*Mpo*^−/−^ animals (Figure S14A). Classical beiging markers like CITED-1 or P2RX5 and thermoregulatory markers, particularly MTS-1 and CIDEA, were upregulated in PVAT of *Lep*^−/−^*Mpo*^−/−^ mice compared to obese *Lep*^−/−^ mice or lean controls (Figures S14B–S14I). Mechanistically, expression of the catalytically active β1 subunit of soluble guanylyl cyclase (sGC-β1), a protective inductor of adipose tissue beiging in obesity,^20^ was significantly elevated in PVAT of HFD-fed *Mpo*^−/−^ and *Lep*^−/−^*Mpo*^−/−^ mice (Figures 5G–5J). These data indicate that MPO exerts a profound impact on adipocyte beiging in PVAT in obesity and suggest reduced sGC-β1 expression as a potential mechanism.

### MPO alters paracrine secretion of vasoactive mediators in PVAT

**Figure 6:**
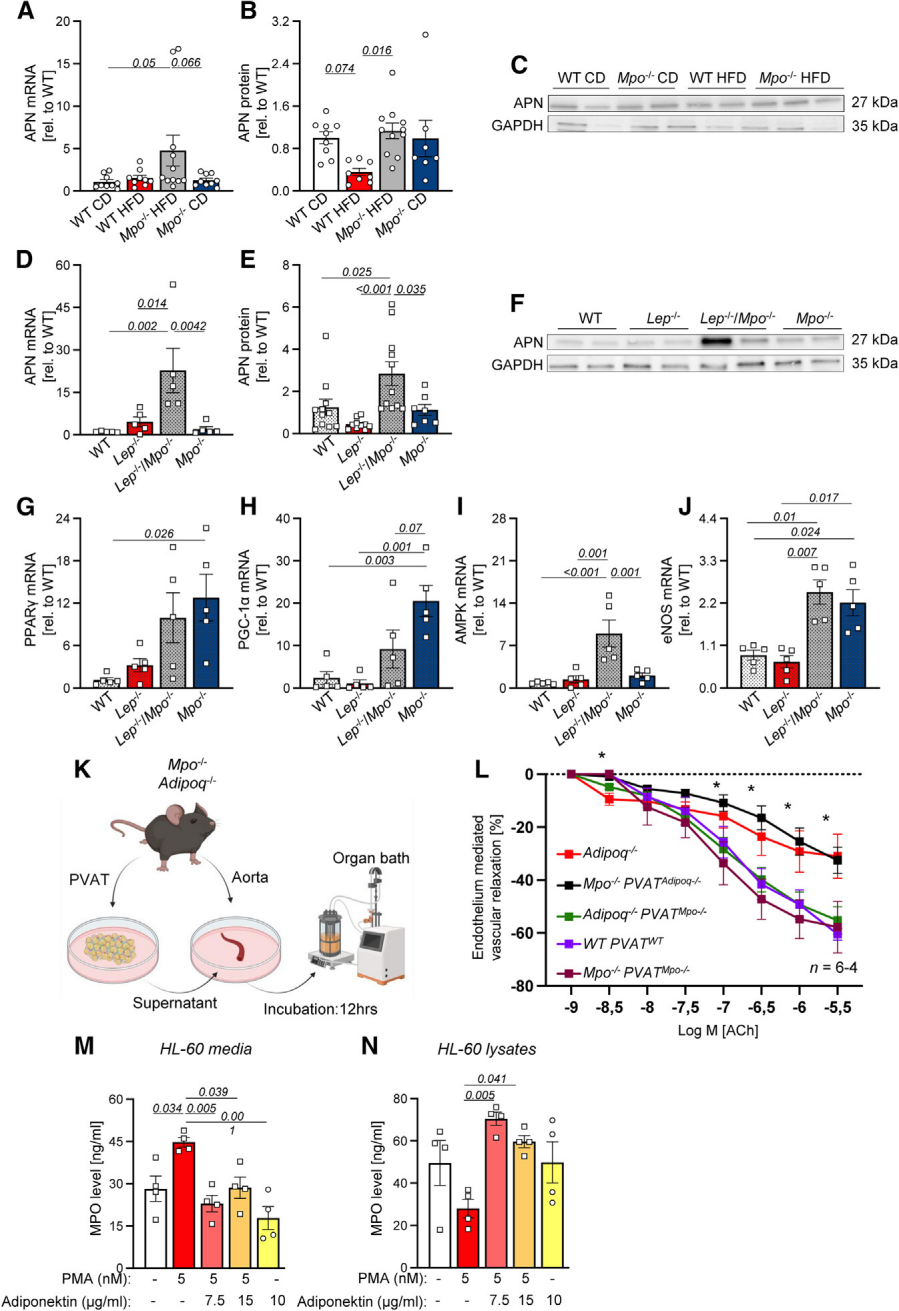
MPO impairs endothelial function by impacting paracrine activity of perivascular adipose tissue APN mRNA and protein expression and representative immunoblots of APN and GAPDH of DIO (A–C) and GIO (D–F) PVAT. mRNA expression of PPARγ, PGC-1α, AMPK, and eNOS in the PVAT of GIO mice (G–J). GAPDH was used as loading control. PVAT was cultivated for 12 h in DMEM. Isolated aortic rings were incubated with the indicated PVAT supernatants, and organ bath experiments were performed (K). Acetylcholine-depended endothelium-mediated vascular relaxation of isolated aortic rings of *Adipoq*^−/−^ mice, aortic rings of *Mpo*^−/−^ mice incubated with A*dipoq*^−/−^ PVAT (*Mpo*^−/−^ PVAT^*Adipoq−/−*^) vice versa, and respective controls (L). MPO protein levels of phorbol 12-myristate 13-acetate (PMA)- and APN-treated PMN-like HL-60 cells within the supernatant (M) and in the cells (N). Data are presented as mean ± SEM. Statistical significance was determined by ordinary two-way ANOVA (A–D, G–J, and L) or by ordinary one-way ANOVA followed by Tukey’s multiple comparison test (M and N). For (L), significances are shown for *Mpo*^−/−^*PVAT*^*Adipoq−/−*^ vs. *Adipoq*^−/−^*PVAT*^*Mpo−/−*^: ∗*p* < 0.05. *n* = as indicated. APN, adiponectin; GAPDH, glyceraldehyde 3-phosphate dehydrogenase; PPARγ, peroxisome proliferator-activated receptor gamma; PGC-1α, peroxisome proliferator-activated receptor gamma coactivator 1-alpha; AMPK, AMP-activated protein kinase; eNOS, endothelial nitric oxide synthase; *Mpo*^−/−^, MPO-deficient; *Adipoq*^−/−^, adiponectin-deficient.

Given that endothelial dysfunction and PVAT inflammation were attenuated by MPO deficiency in obesity, we next explored the expression of paracrine regulators potentially contributing to endothelial function. Transcript and protein expression of APN, a well-known modulator of endothelial function, were elevated in PVAT of obese *Mpo*^−/−^ mice compared to obese WT animals in the DIO (Figures 6A–6C) and in the GIO (Figures 6D–6F) model. In line with this finding, peroxisome proliferator-activated receptor gamma (PPARγ), a key regulator of APN expression,^21^ showed enhanced mRNA expression in *Mpo*^−/−^ mice, which was trend-wise elevated in obese *Mpo*^−/−^ animals compared to lean controls (Figure 6G). Likewise, the expression of PPARγ coactivator 1α (PGC-1α), a positive regulator of mitochondrial biogenesis and BAT differentiation, was upregulated in *Mpo*^−/−^ mice and numerically in obese *Mpo*^−/−^ animals as well (Figure 6H). APN mediates its vasoprotective effects by adenosine monophosphate kinase (AMPK)-induced stimulation of endothelial NO synthase (eNOS). MPO deficiency significantly increased AMPK and eNOS mRNA expression in the GIO model (Figures 6I and 6J). Of note, MPO deficiency elevated PPARγ, PGC-1α, and eNOS expression in PVAT also in lean mice (Figures 6G, 6H, and 6J). To demonstrate that MPO’s effect on paracrine activity of PVAT—and in particular on APN secretion—contributes profoundly to a worsened vascular function, aortic rings of APN-deficient mice (*Adipoq*^−/−^) mice were incubated with supernatant from cultured primary PVAT adipocytes of *Mpo*^−/−^ mice and vice versa and subjected to organ bath investigation (Figure 6K). Acetylcholine stimulation revealed impaired endothelial function of *Adipoq*^−/−^ aortic rings similar to observations made in aortic rings isolated from obese mice (Figure 6L, red). Comparably, incubation of *Mpo*^−/−^ aortic rings with supernatant from *Adipoq*^−/−^ PVAT led to an impaired endothelial relaxation (Figure 6L, black). In contrast, endothelial dysfunction of *Adipoq*^−/−^ aortic rings was rescued when incubated with supernatant from *Mpo*^−/−^ PVAT (Figure 6L, green) and was comparable to WT and *Mpo*^−/−^ aortic rings incubated with PVAT from WT or *Mpo*^−/−^ mice, respectively (Figure 6L, violet, brown).

Anti-inflammatory effects of APN on leukocytes were tested by stimulating neutrophil-like HL-60 cells with APN *in vitro*. Interestingly, APN treatment led to a significant reduction in MPO release (Figure 6M) and to enhanced MPO retention within the cells (Figure 6N).

These data suggest MPO to have a crucial impact on PVAT secretome and endothelial function, especially by altered APN expression. Noteworthily, this crosslink seems to be independent of circulatory or systemic inflammatory effects.

### MPO induces adipocyte whitening, inflammatory activation, and reduced APN secretion of adipocytes *in vitro*

**Figure 7:**
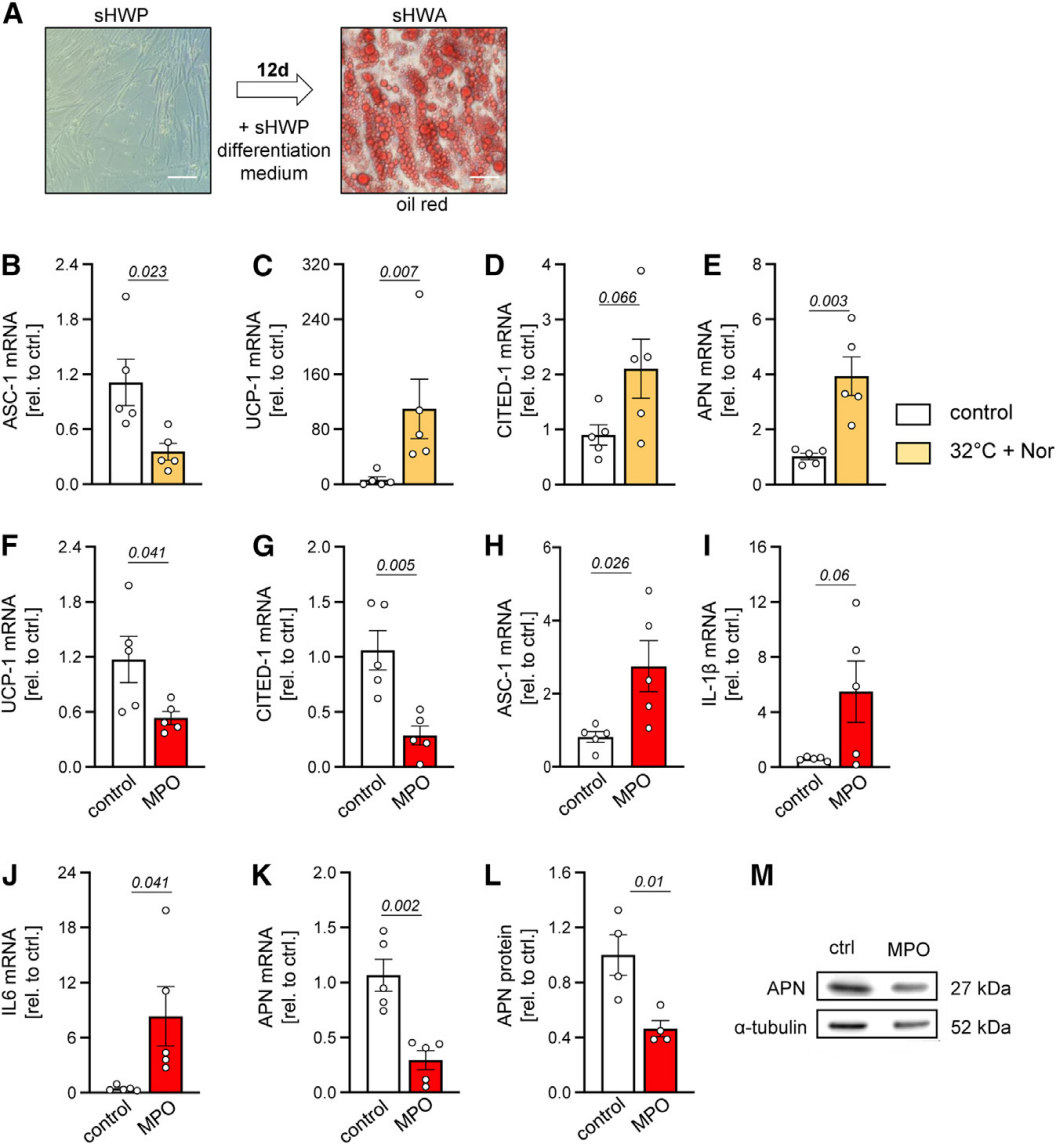
MPO changes adipocyte phenotype, cytokine, and adipokine expression *in vitro* Subcutaneous human white preadipocytes (sHWPs) were differentiated to adipocytes (sHWAs), identified by excessive lipid vacuole formation (A, red circles in oil red O staining). Expression of ASC-1, UCP-1, CITED-1, and APN mRNA of sHWA incubated at 32°C and stimulated with noradrenaline (Nor) for BAT induction and control cells incubated at 37°C without Nor treatment (B–E). UCP-1, CITED-1, ASC-1, IL-1β, IL-6, and APN mRNA expression of MPO/H_2_O_2_-treated or control-treated beiged sHWA (F–K). APN protein expression of MPO/H_2_O_2_- or control-treated sHWA (L and M). α-tubulin was used as loading control. Data are presented as mean ± SEM. Statistical significance was determined by unpaired Student’s t test; for (C), a non-parametric Mann-Whitney test was used. *n* = as indicated. ASC-1, Asc-type amino acid transporter 1; UCP-1, uncoupling protein 1; CITED-1, Cbp/p300-interacting transactivator 1; APN, adiponectin; IL-1β, interleukin 1β; IL-6, interleukin 6; BAT, brown adipose tissue.

To translate our findings and investigate specific effects of MPO on the adipocyte phenotype as well as cytokine and adipokine expression, subcutaneous human white preadipocytes (sHWPs) were differentiated to mature adipocytes (sHWAs) *in vitro*. Maturation was confirmed by the presence of intracellular lipid vacuoles (Figure 7A). Beiging was induced by noradrenaline and incubation at 32°C for 4 h. Beiged sHWAs showed markedly reduced mRNA transcription of the WAT marker ASC-1 and significantly higher mRNA expression of the BAT markers UCP-1 and APN and, numerically, CITED-1 as compared to control cells incubated at 37°C (Figures 7B–7E). Importantly, incubation with MPO and its substrate H_2_O_2_ significantly reduced adipocyte beiging reflected by reduced expression of UCP-1 and CITED-1 and an increase in ASC-1 mRNA expression (Figures 7F, 7G, and 7H). Furthermore, MPO stimulation enhanced numerically IL-1β and significantly IL-6 expression compared to control treatment (Figures 7I and 7J). APN mRNA and protein expression were significantly attenuated by MPO treatment (Figures 7K, 7L, and 7M). These results suggest a direct impact of MPO on adipocyte whitening, pro-inflammatory cytokine production, and the expression of endothelium-relevant adipokines in the context of obesity.

### MPO deficiency elevates energy consumption in obesity

We next analyzed overall metabolic effects of MPO in obese mice. As a marker of long-term blood sugar levels, we determined HbA1c levels in GIO mice, which was reduced in *Lep*^−/−^/*Mpo*^−/−^ mice compared to *Lep*^−/−^ animals (Figure S15A). Accordingly, insulin tolerance was increased upon MPO deficiency in *Lep*^−/−^ mice as indicated by blood glucose measurements (Figure S15B). Metabolic characterization using PhenoMaster cages revealed a reduction in oxygen consumption (Figures S15C and S15D) and carbon dioxide production (Figures S15E and S15F) in *Lep*^−/−^ mice, which was significantly attenuated in *Lep*^−/−^/*Mpo*^−/−^ animals during resting phase. Raw oxygen consumption and carbon dioxide production data per mouse are shown in Figures S16A and S16D. Whereas food uptake was not different between genotypes (Figure S15G), a trend of augmented water uptake was observed in *Lep*^−/−^ mice as compared to *Lep*^−/−^/*Mpo*^−/−^ littermates (Figure S15H). Analysis of covariance did not show any significant effect of body weight on the metabolic parameters (Figure S16E). mRNA expression analyses of additional adipose tissue types revealed an increase of adipocyte beiging markers P2RX5 and CITED-1 in WAT of obese *Mpo*^−/−^ mice in the GIO and DIO model, respectively, and lower expression of the WAT marker ASC-1 in GIO mice. UCP-1 expression was significantly upregulated in BAT of obese MPO-deficient DIO mice (Figure S17). In WAT of HFD-fed *Mpo*^−/−^ mice, AMPK and eNOS gene expression were upregulated compared to obese WT mice. Congruently, eNOS expression was elevated in BAT *of Mpo*^−/−^ mice after HFD (Figure S18A). Plasma levels of APN were investigated in both models without showing significant differences. (Figure S18B).

## Discussion

PVAT is an immune cell-hosting, endocrine organ in close proximity to the arterial wall, which directly influences vascular function in various pathologies e.g., atherosclerosis, coronary artery disease, and aortic aneurysm formation.^22^^,^^23^^,^^24^ Herein, we demonstrate that the neutrophil-derived heme-peroxidase MPO impacts on vascular function in obesity by changing paracrine and phenotypic properties of PVAT. We provide evidence of MPO affecting vascular function “from outside to inside” in complement to the common paradigm of an “inside-to-outside” action of circulating MPO.

In a cohort of 33 patients with obesity, we found weight reduction by bariatric surgery to be accompanied by a significant improvement of endothelial function as measured by FMD (Figure 1G). Similar observations were made by Gokce et al., indicating a link between body weight and vascular function.^25^ Furthermore, levels of MPO significantly declined upon body weight reduction. Improvement of endothelial function inversely correlated with circulating MPO levels, suggesting MPO as a biologically relevant mediator of adipose tissue metabolism and vascular function in obesity (Figure 1H). Multiple contributing roles of MPO in aortic pathologies have been reported^15^^,^^26^^,^^27^; however, effects of MPO on abluminal parts of the vessel wall and in particular on PVAT have not been investigated so far. MPO exists in both free and membrane-bound forms, with the latter bound to endothelial surfaces, platelets, and erythrocytes.^17^^,^^28^^,^^29^ Our findings, along with previous studies, suggest that free MPO levels correlate with membrane-bound MPO in coronary artery disease.^30^ Therefore, we anticipate that obesity may not significantly alter the ratio of free-to-bound MPO, though this remains an intriguing question for future research.

Beige adipocytes are characterized by a high potential of thermogenesis via energy dissipation, thus reducing cellular lipid storage.^11^ In this context, we observed an attenuated weight gain in both, a dietary and a genetic murine obesity model upon MPO deficiency (Figures 2B and 2C). Similar results were reported by Piek et al., showing that weight gain of mice was reduced after HFD when treated with the MPO inhibitor AZM198 for 16 weeks, although underlying mechanisms were not investigated.^26^

Obesity increased MPO plasma and PVAT levels and was accompanied by enhanced myeloid cell frequency, which was curbed by MPO deficiency. Pro-inflammatory cytokines were elevated in PVAT of MPO-competent obese mice and in MPO-treated adipocytes, whereas MPO deficiency attenuated IL-1β, TNF-α, and CCL-2 expression *in vivo* (Figures 4A–4C). These data are in line with previous findings indicating strong pro-inflammatory properties of MPO either by enhanced cytokine production^31^ or by attracting neutrophils via physical forces.^32^ In addition, nitrosative stress caused by MPO’s catalytic activity has been linked to endothelial damage and atherosclerosis development.^33^

Obesity accelerates PVAT dysfunction by adipocyte whitening and increased pro-inflammatory cytokine secretion.^11^ PVAT in obese *Mpo*^−/−^ mice exhibited adipocyte beiging as indicated by upregulation of UCP-1. This finding was confirmed by an altered adipocyte morphology with smaller lipid vacuoles combined with a downregulation of the WAT marker ASC-1 and simultaneous upregulation of beiging markers and thermoregulatory genes (Figure 5). Furthermore, expression of UCP-1 and CITED-1 was diminished in adipocytes after incubation with MPO *in vitro*, confirming MPO to inhibit adipocyte beiging (Figure 7). In summary, MPO fosters a dysfunctional adipocyte phenotype and impedes fat utilization, ultimately limiting cardiovascular health.^34^

In line with our findings, prevention of adipocyte beiging is linked to increased inflammatory activation of PVAT, leading to pathological vascular remodeling.^24^ Here, we demonstrate a strong interdependency between PVAT inflammation and MPO-mediated phenotypic changes impeding PVAT beiging in obesity. We show that PVAT beiging in obese mice is suppressed by MPO via inhibition of sGC-β1, a crucial inductor of WAT beiging via cyclic guanosine monophosphate (cGMP) production.^35^ Furthermore, MPO treatment of thoracic aortas decreased sGC-β1 expression and function by sGC oxidation^16^^,^^20^ (Figures 5G–5J).

APN, an adipokine produced by PVAT, has been shown to essentially maintain endothelial function by induction of eNOS via the AMPK pathway.^36^^,^^37^ MPO contributes to PVAT dysfunction in obesity in an APN-dependent manner, leading to pro-contractile and deleterious adipokine signaling, ultimately impairing endothelial function.

When aortic rings of *Adipoq*^−/−^ mice were incubated with supernatant of PVAT from *Mpo*^−/−^ mice, endothelial function was rescued, whereas incubation of *Mpo*^−/−^ aortic rings with supernatant from *Adipoq*^−/−^ mice significantly worsened endothelial function (Figures 6K and 6L). These findings verified that altered APN expression of *Mpo*^−/−^ PVAT essentially contributes to improved endothelial function in obesity. Furthermore, *in vitro* data revealing decreased APN gene expression of human adipocytes upon MPO stimulation fostered these findings. APN expression is induced by PPARγ, which was upregulated on gene expression levels in *Mpo*^−/−^ mice^38^ (Figure 6G). Mechanistically, the pro-inflammatory cytokine TNF-α and reactive oxygen species (ROS), which were increased in MPO-competent obese mice, have been described to suppress APN^39^^,^^40^ and might mediate MPO’s effects on APN expression. Furthermore, increased expression of CCL-2 in MPO-deficient obese PVAT might further reduce leukocyte infiltration leading to attenuated TNF-α and ROS expression and subsequent elevated APN secretion.

Given that inflammatory stimuli suppress the production of APN,^41^ and that APN exhibits anti-inflammatory effects by reducing MPO release from PMN *in vitro*, this suggests a potential vicious cycle: inflammation in obesity reduces APN expression, leading to higher MPO release by PMN in PVAT, which in turn further reduces APN secretion and exacerbates endothelial dysfunction. APN levels’ changing only in PVAT and not systemically underscores the predominantly paracrine nature of this mechanism.

In line with FMD and MPO measurements obtained from bariatric patients, organ bath experiments confirmed obesity-induced endothelial dysfunction to be attenuated dramatically in *Mpo*^−/−^ mice in both obesity models, indicating that this effect is for the most part independent of altered leptin signaling in *Lep*^−/−^ mice (Figures 2F and 2G).

We observed MPO deficiency to directly improve metabolism and insulin tolerance. Although energy consumption and metabolism underlie complex regulative mechanisms, our data suggest overall beiging of adipose tissues by enhanced expression of thermoregulatory genes and BAT markers in MPO-deficient animals to contribute to this effect.^42^ The ability of beige adipocytes to perform non-shivering thermogenesis might potentially account for the mildly reduced body weights observed in MPO-deficient obese animals. It is also conceivable that MPO directly oxidizes UCP-1 by the production of HOCl, as oxidative modification of UCP-1 by ROS represents a regulatory mechanism.^43^^,^^44^ However, further investigation is needed to elucidate the molecular effects underlying the increased beiging of adipose tissue in the context of MPO deficiency.

A preliminary preclinical study with a 2-week administration of the MPO inhibitor 4-ABAH already showed an improvement in PWV in obese mice, which underscores the clinical relevance of our findings (Figures S6D–S6E). In addition, recent results from a clinical phase 2a study (Safety and Tolerability Study of AZD4831 in Patients With Heart Failure, SATELLITE) revealed therapy with the MPO inhibitor AZD4831 to be well tolerated and safe in heart failure patients with preserved ejection fraction.^45^ Thus, pharmacotherapeutic MPO inhibition in humans is possible and might be tested in a translational approach to prove beneficial effects on endothelial function and atherosclerosis development in patients with obesity.

### Limitations

Patient data are limited to circulating cytokine and MPO levels with respect to body weight. PVAT of those patients has not been investigated due to its limited accessibility. Given that a direct impact of MPO at the endothelial side of the vessel is well described, these effects could not be fully excluded when investigating endothelial function in our models. Nonetheless, co-incubation experiments of aortic rings and PVAT supernatant as well as *in vitro* data demonstrated a strong effect of MPO on adipocytes independent from luminal events. The absence of lean body mass measurements in our metabolic study (Figures S15 and S16) is a limitation, as normalization of metabolic rates to body mass would have provided additional precision for data interpretation.^46^ Finally, obesity influences various signaling pathways and vasoactive mediators next to MPO that have not been investigated and might contribute to PVAT phenotype, expression profile, and endothelial dysfunction.

### Conclusion

MPO, a heme-peroxidase predominantly expressed by neutrophilic granulocytes, accumulates in PVAT as part of an obesity-related adipose tissue inflammation and exerts detrimental effects on the vasculature via alteration of PVAT adipocyte signature and expression profile. MPO inhibits adipocyte beiging as a protective mechanism from obesity-induced endothelial dysfunction and furthermore fosters immune cell frequency, ROS formation, and pro-inflammatory cytokine production. In addition, MPO dampens APN release from PVAT, contributing to endothelial and vascular dysfunction in an outside-to-inside directed manner.

## Resource availability

### Lead contact

Further information and requests for resources and reagents should be directed to and will be fulfilled by the lead contact, Martin Mollenhauer, martin.mollenhauer@uk-koeln.de.

### Materials availability

(1) Plasmids were not generated in this study.
(2) No new mouse lines were generated in this study.
(3) Crossbred *Lep*^−/−^ (B6.Cg-Lepob/J) and *Mpo*^−/−^ (B6.129X1-Mpotm1Lus/J) mice were obtained from and are available at The Jackson Laboratory.
(4) This study did not generate new unique reagents.
(5) To our knowledge, there are no restrictions to the availability of reagents used in this study.

### Data and code availability

• No datasets of standardized data type such as RNA-seq, metabolomics, or proteomics were generated in this study.
• This study does not report custom computer codes.
• Any additional information required to reanalyze the data reported in this work paper is available from the lead contact upon request.

## Acknowledgments

The authors would like to thank Christina Vosen, Sharon Weingarten, Katharina Tinaz, Nadja Klein, and Simon Grimm for excellent technical support. This work was supported by the 10.13039/501100001659Deutsche Forschungsgemeinschaft (GRK 2407 [360043781] to M.M., H.W., D.M., and S.G.; SFB TRR259 [397484323] project A04 to H.W. and S. Baldus, project B05 to M. Adam, project A08 to A.P. and S.H., and project C03 to M.M.; MO 3438/2-1 to M.M.; and HO 5279/2-1 to F.F.H.), the 10.13039/501100009983Center for Molecular Medicine Cologne B 12 to H.W. and M.M., the Neven-DuMont Foundation to H.W., and the Koeln Fortune Program (344/2019 to S. Braumann, 363/2020 to F.S.N., and 248/2021 to A.H.).

## Author contributions

A.H., J.S., and M.M. designed and conducted *in vitro* and *in vivo* experiments, analyzed the data, and wrote the manuscript. P.P., R.H., P.v.S., S. Braumann, S. Baldus, and M. Ahdab performed the human study and analyzed the data. H.N., F.S.R.P., and F.F.H. performed *in vivo* experiments and corresponding data analyses. J.C.B. and J.A. performed the metabolic cage study and analyzed the corresponding data. A.P. and S.H. helped with the BAT and WAT and the fatty tissue inflammation analyses. M.L., M. Adam, F.S.N., and H.G. performed *ex vivo* organ collection and protein analyses. M.L., D.M., and S.G. performed histological stainings.

## Declaration of interests

The authors declare no competing interests.

## Declaration of generative AI and AI-assisted technologies in the writing process

The authors did not use any generative AI for preparation of this manuscript.

## STAR★Methods

### Key resources table

**Table:** 

REAGENT or RESOURCE	SOURCE	IDENTIFIER
**Antibodies**		

Rat Anti-Ly-6C Monoclonal Antibody, FITC Conjugated, Clone AL-21	BD Biosciences	Cat# 553104, RRID:AB_394628
CD115 (c-fms) Monoclonal Antibody (AFS98), PerCP-eFluor™ 710, eBioscience	Thermo Fisher Scientific	Cat# 46-1152-82, RRID:AB_10597740
APC anti-mouse Ly-6G	BioLegend	Cat# 127614 RRID:AB_2227348
Brilliant Violet 711(TM) anti-mouse CD45	BioLegend	Cat# 103147 RRID:AB_2564383
Mouse Anti-Mouse CD64 a and b Alloantigens Antibody, Alexa Fluor 647 Conjugated	BD Biosciences	Cat# 558539 RRID:AB_647120
APC/Cyanine7 anti-mouse/human CD11b Antibody	BioLegend	Cat# 101226 RRID:AB_830641
PE anti-mouse NK-1.1	BioLegend	Cat# 108708 RRID:AB_313395
PE anti-mouse TER-119/Erythroid Cells	BioLegend	Cat# 116208 RRID:AB_313709
PE anti-mouse CD90.2 (Thy1.2)	BioLegend	Cat# 105308 RRID:AB_313179
PE anti-mouse Ly-6G	BioLegend	Cat# 127608 RRID:AB_1186099
PE anti-mouse/human CD45R/B220	BioLegend	Cat# 103208 RRID:AB_312993
Brilliant Violet 605(TM) anti-mouse CD19	BioLegend	Cat# 115539 RRID:AB_11203538
Adiponectin Polyclonal Antibody	Thermo Fisher Scientific	Cat# PA1-054 RRID:AB_325789
Anti-UCP1 antibody	Abcam	Cat# ab155117 RRID:AB_2783809
Guanylate Cyclase β1 subunit (soluble) Polyclonal Antibody	Cayman Chemical	Cat# 160897 RRID:AB_10080042
Rabbit Anti-GAPDH Monoclonal Antibody, Unconjugated, Clone 14C10	Cell Signaling Technology	Cat# 2118, RRID:AB_561053
Anti-Rabbit IgG (whole molecule)-Peroxidase antibody produced in goat	Sigma-Aldrich	Cat# A0545 RRID:AB_257896
Alexa Fluor(R) 594 anti-mouse Ly-6G	BioLegend	Cat# 127636 RRID:AB_2563207
ICAM-1 Monoclonal Antibody (3E2B)	Thermo Fisher Scientific	Cat# MA5405 RRID:AB_223595
Purified anti-mouse CD68	BioLegend	Cat# 137002, RRID:AB_2044004
Goat Anti-Goat IgG (H + L) Antibody, Alexa rFluor 594 Conjugated	Thermo Fisher Scientific	Cat# A21468, RRID:AB_10563772
Chicken anti-Mouse IgG (H + L) Cross-Adsorbed Secondary Antibody, Alexa Fluor™ 488	Thermo Fisher Scientific	Cat# A-21200, RRID:AB_2535786
Cy3-AffiniPure Goat Anti-Armenian Hamster IgG (H + L) (min X Bov Sr Prot)	Jackson ImmunoResearch Labs	Cat# 127-165-099, RRID:AB_2338988
Alexa Fluor® 594 Rat IgG2a, κ Isotype Ctrl	BioLegend	Cat# 400555, RRID:AB_3096997
Armenian Hamster IgG Isotype Control (eBio299Arm), eBioscience	Thermo Fisher Scientific	Cat# 14-4888-81, RRID:AB_470128
Goat IgG Isotype Control	Thermo Fisher Scientific	Cat# 02–6202, RRID:AB_2532946
Mouse IgG Isotype Control	Thermo Fisher Scientific	Cat# 31903, RRID:AB_10959891
Anti-Nitrotyrosine	Avantor	CAT# BIRBORB25786-1 RRID: N/A

**Biological samples**		

Human blood samples	University Hospital Hamburg-Eppendorf (PV3615)	N/A

**Chemicals, peptides, and recombinant proteins**		

Recombinant Human Adiponectin	PreproTech	CAT# 450 24
Myeloperoxidase Inhibitor I (4-ABAH)	Sigma-Aldrich	CAT# 475944-1GM

**Critical commercial assays**		

CardioMPO-Assay Kit	Cleaveland HearthLab	CAT# C133
MPO-ELISA	Hycult biotech	CAT# HK210-01
Direct-Zol RNA MiniPrep Plus kit	Zymo-Research	CAT# R2072
QuantiTect Reverse Transcription Kit	Qiagen	CAT# 205311
GoTaq Master Mix	Promega	CAT# M7122
TrueVIEW autofluorescence quenching Kit	Vector Laboratories	CAT# SP-8400-15

**Experimental models: Cell lines**		

Primary human white preadipocytes	Promocell	CAT# C-12735
HL-60 cells	N/A	RRID:CVCL_0002

**Experimental models: Organisms/strains**		

Mus Musculus C57BL6/J Mpo^−/−^ (B6.129X1-Mpotm1Lus/J)	Jackson Laboratory	JAX:004265
Mus Musculus C57BL6/J Lep^−/−^ (B6.Cg-Lepob/J)	Jackson Laboratory	JAX:000632
Mus Musculus C57BL6/J Adipoq^−/−^ (B6; 129-Adipoqtm1Chan/J)	Jackson Laboratory	JAX:008195

**Oligonucleotides**		

For all used primers see at S4	This paper	N/A

**Software and algorithms**		

Brachial Analyzer software (4.1.3)	Medical imaging application LCC	https://mia-llc.com/services/brachial.htm
FusionCapt Advance FX7 (16.06)	Vilber Lourmat	http://www.vilber.de/produkte/geldokumentation/kombisysteme/fusion-fx7-advanced/
ImageJ (1.53a)	NHI	https://imagej.net/ij/download.html
Vevo 7 Vasc, Fujifilm	Visual sonics	https://www.visualsonics.com/product/software/vevo-vasc-software
GraphPad Prism (8.4.0)	Graphpad Software)	https://www.graphpad.com/features
SPSS statistics (29.0.0)	IBM	https://www.ibm.com/de-de/products/spss-statistics

### Experimental model and study participant details

#### Human studies

50 patients suffering from obesity with a BMI >37 kg/m^2^ admitted for gastric bypass surgery or sleeve gastrectomy were enrolled in the study. 17 patients were lost to follow-up or retracted study consent. 14 lean patients served as control cohort. Exclusion criteria were major surgical procedures, malignant diseases or other limiting conditions in the past medical history. Patients pre-treated with heparins or known intolerance to nitroglycerin were excluded from the study. The study protocol was approved by the Ethics Committee of the Hamburg Medical Association (PV3615) and the Ethics Commission of Cologne University’s Faculty of Medicine (19-1585) and conducted in compliance with the declaration of Helsinki. All patients gave written informed consent prior to enrollment into the study. 17 obese patients were lost to follow-up or retracted study consent. Further detailed information on the study participants can be found in Table S1. Information on ancestry, gender, ethnicity and socio-economic status were not collected in this study.

#### Animal studies

Studies were performed within a genetic and a dietary obesity mouse model (all C57BL6/J background). For the genetically inherited obesity mouse model (GIO), leptin-deficient (*Lep*^−/^) mice were crossbred with MPO-deficient mice (*Lep*^−/−^/*Mpo*^−/−^). *Lep*^−/−^, *Mpo*^−/−^, and *Adipoq*^−/−^ were obtained from Jackson Laboratory (Bar Harbor, USA). *Lep*^−/−^ (B6.Cg-Lepob/J) and *Mpo*^−/−^ (B6.129X1-Mpotm1Lus/J) mice were obtained from and are available at The Jackson Laboratory. In the dietary induced obesity mouse model (DIO), 8 weeks-old WT and *Mpo*^−/−^ mice were fed a 60% high-fat diet *ad libitum* (HFD; Ssniff-DIO 60kJ %, D12492E15742-347, 10mm pellets, γ-irradiated) for 12 weeks. The age of the GIO mice at organ collection was 16–20 weeks, that of the DIO and 4-ABAH mice 20–22 weeks. Chow-diet fed WT (WT CD, Ssniff- V1534-703, 10mm pellets, γ-irradiated) and *Mpo*^−/−^ (*Mpo*^−/−^ CD) littermates at the corresponding age group served as controls. The mice were randomly assigned to groups by cage. Both sexes were used for DIO, GIO and the 4-ABAH treated mice. The weights of the DIO and GIO mice can be found in Figures 2B and 2C. All mice were group housed under clean laboratory conditions in NexGen Mouse 500 cages (Allentown Inc., Allentown, USA) under a 12/12 light cycle and were provided with water *ad libitum*. Apart from the investigations and interventions described here, no further medication or experiments were carried out on the mice. All animal studies were conducted to relevant regulatory standards and approved by the local authorities (Ministry for Environment, Agriculture, Conservation and Consumer Protection of the State of North Rhine-Westphalia: State Agency for Nature, Environment and Consumer Protection (LANUV, NRW, Germany with the authorization number: 81–02.04.2018.A216) and by the University of Cologne Animal Care and Use Committees. All experiments were performed blinded.

#### Cell culture

Primary human white preadipocytes (sHWP, C-12735, Promocell, Heidelberg, Germany) were cultured in preadipocyte growth medium (C-27410, Promocell) supplemented with 1% penicillin and streptomycin at 37°C and 5% CO_2_. On day 3, sHWP reached confluency and preadipocyte differentiation medium (C-27436, Promocell) was added for 3 days. Subsequently, cells were kept in adipocyte nutrition medium (C-27438, Promocell), reaching full differentiation within 12 days. To induce beiging of fully differentiated subcutaneous human white adipocytes (sHWAs), cells were incubated at 32°C and stimulated with 1 μM noradrenaline for 4 h. 50,000 cells were treated with 1 μg/mL MPO and 4 μM H_2_O_2_ in adipocyte nutrition medium containing 3% BSA for 12 h to investigate MPO’s effect on sHWA beiging, APN and interleukine expression.

HL-60 cells were cultured in a 12-well plate in RPMI (Thermo Fisher, Waltham, USA) containing 20% fetal bovine serum and 1% penicillin/streptomycin. 50,000 cells were plated per well and treated with APN 7.5μg/L or 15μg/ml with and without phorbol 12-myristate 13-acetate (PMA; 5 nM) for 60 min at 37°C. Subsequently, media and cells were separately collected for further analysis. The controls remained untreated. Mycoplasma tests were conducted regularly. The cells ordered by Promocell and the HL-60 were not re-authenticated. The experiments were carried out according to the STAR Methods described above. Blinding was conducted in these studies in accordance with the respective treatment approach.

### Method details

#### Human blood sampling

Test subjects were advised not to smoke for 6 h prior to blood sampling and assessment of vascular function. Blood samples were taken immediately before flow-mediated dilation measurements and then centrifuged at 4000 rpm for 10 min. EDTA-plasma was collected and stored at −80 °C at both research sites (Hamburg and Cologne, Germany) and MPO level were measured after 1 freeze-thaw cycle, respectively. Follow-up measurements were performed three months after surgery.

#### Flow-mediated dilation measurement

A Siemens Sonoline ultrasound device with a 12 MHz linear array transducer was used to examine vascular function by measuring the flow-mediated dilation (FMD) according to the guidelines of the International Artery Reactivity Task Force.^27^^,^^47^ In brief, a two-dimensional sequence of the brachial artery was obtained at baseline and after arterial occlusion by inflating a blood pressure cuff to at least 50 mmHg above systolic blood pressure. Velocity time integral (VTI) was measured during cuff deflation using a pulsed wave doppler. One minute after cuff deflation, diameter of the brachial artery was measured at the initial position. FMD was defined as percentage increase in post-stimulus diameter from baseline diameter using the Brachial Analyzer software (Medical imaging application LLC, version 4.1.3).

#### MPO, LDL and hsCRP plasma-levels

MPO plasma levels were measured with the CardioMPO-Assay Kit (Cleveland HeartLab, Cleveland, USA). High-sensitive C-reactive protein (hsCRP) was measured with the Roche/Hitachi P Analyzer. Low-density lipoprotein (LDL) levels were determined by the central clinical chemistry laboratory of the University Hospital Hamburg-Eppendorf.

#### MPO inhibitor administration

For pharmacological MPO inhibition experiments, the MPO inhibitor 4-ABAH was administered to DIO mice every other day during the last two weeks of feeding by intraperitoneal injection. 4-ABAH (Sigma Aldrich, St. Louis, USA) was diluted in sterile dimethyl sulfoxide (DMSO, Sigma Aldrich, St. Louis, USA) according to the manufacturer's instructions. The final concentration was 4 mg/ml 4-ABAH in 5% DMSO in sterile isotonic sodium chloride. In accordance with previous studies, we used a dose of 20 mg 4-ABAH per kg mouse weight. The control group received 5% DMSO diluted in NaCl.

For organ collection, mice were anesthetized by isoflurane inhalation (Isofluran-Piramal®, Piramal Critical Care, Voorschoten, The Netherlands; 5 % vol/vol for induction and 2 % vol/vol for maintenance of anaesthesia) and s.c. injection of buprenorphine (TEMGESIC®, Indivior Europe Limited, Dublin, Ireland; 0.1 mg/kg BW) followed by cardiac exsanguination. PVAT collection is shown in Figure S3. WAT was harvested from visceral fat depots and BAT from interscapular fat depots.

#### Metabolic characterization

The GIO mouse model was used for metabolic characterization using Phenomaster cages and for the assessment of insulin resistance. The Phenomaster cage (TSE, Bad Homburg, Germany) contains an indirect colorimetric measurement, which allows the assessment of CO_2_ generation and O_2_ consumption. Food and water intake is measured by a weighing system. The insulin resistance test was performed by intraperitoneal insulin injection (0.75 IE/kg) followed by blood glucose measurements at 0, 15, 30 and 60 min. Blood was collected from the tail vein. HbA_1C_ levels were determined by the central clinical chemistry laboratory of the University Hospital Cologne. Before the experiments, the animals were placed in the metabolic cages two days prior to allow an acclimatization period.

#### Flow cytometry

PVAT was harvested and minced in digestion buffer containing 450 U/ml collagenase I (Merck Sigma-Aldrich, Waltham, USA), 125 U/ml collagenase XI (Merck Sigma-Aldrich), 60 U/ml hyaluronidase I-S (Merck Sigma-Aldrich), 60 U/ml DNase I (Merck Sigma-Aldrich), HEPES buffer (Thermo Fisher Scientific, Waltham, USA) and PBS (Thermo Fisher Scientific). Tissue digestion was performed for 1 h at 37°C on a shaker at 450 rpm. Subsequently, samples were filtered twice with staining buffer (0.5% BSA in PBS) through a 50 μm and 30 μm nylon cell strainer (Corning, Corning, USA) to obtain a single cell suspension. Cells were centrifuged at 350 x g at 4°C for 7 min and stained with antibodies diluted 1:100 (Table S2) at 4°C for 30 min for myeloid cell characterization. For blood sample preparation, 350 μl of RBC lysis buffer (eBioscience™ 1x RBC Lysepuffer) was added to each sample of 70 μl blood, vortexed and incubated for 3 min. Subsequently, cell lysis was stopped by addition of 4,5 ml PBS. Cell pellets were washed twice, resuspended in 300 μL staining buffer and stained as described before.

The stained cells were washed again with 4 mL staining buffer, the cell pellet was dissolved in 300 μL staining buffer and analyzed with a BD LSRFortessa flow cytometer (BD Biosciences, Madrid, Spain). The flow cytometry data analysis was performed with FlowJo version 10.8.1 (BD Biosciences).

#### Protein quantification

Liquid nitrogen snap-frozen PVAT, WAT and BAT samples were homogenized in Precellys (VWR, Radnor, USA) ceramic kit tubes (1.4 mm, 2.0 mL) with 200 μL ice-cold RIPA lysis buffer (150 mM NaCl, 5 mM EDTA pH 8.0, 50 mM Tris pH 8.0, 1% NP40, 0.5% sodium deoxycholate 0.1% SDS) containing protease and phosphatase inhibitors (Roche, Mannheim, Germany). Supernatants were collected after repetitive vortexing and centrifugation. Total protein concentration was measured by Pierce Bicinchoninic Acid Protein Assay Kit (Thermo Fisher Scientific). For tissue and plasma MPO detection, samples were processed according to the manufacturer’s instructions using the Hycult biotech MPO ELISA (HK210-01, Hycult biotech, Uden, Netherlands). For immunoblotting, equal amounts of total protein lysates were diluted in Laemmli buffer (126mM Tris base, 40% glycerol, 8% sodium dodecyl sulfate, 0.04% bromphenol blue, adjusted to pH 6.8 with HCl) and 10% dithiothreitol, then heated to 97°C for 10 min to ensure complete denaturation and subjected to SDS-PAGE. After electrophoresis, the proteins are transferred to a nitrocellulose membrane by electroblotting. These membranes were then blocked in BSA (1% Tween 20, tris-buffered saline) for 1 h and incubated with respective primary antibodies listed in Table S3 overnight at 4°C. Bound antibodies were visualized using an HRP-linked secondary antibody (Goat Ani-Rabbit IgG Peroxidase antibody, Merck Sigma-Aldrich), followed by a chemiluminescence assay (ECL, Thermo Fisher Scientific) using the fusion FX7 imaging system (Vilber Lourmat, Collegien, France). Protein detection was quantified by optical density using FusionCapt Advance FX7 Software version 16.06 (Vilber Lourmat). Blots are shown in Figure S7. For PVAT, GAPDH was used, while for HWA protein expression alpha-tubulin was implemented as loading control.

CC-chemokine ligand 2 (CCL2), CC-chemokine ligand 4 (CLL4), CC-chemokine ligand 5 (CCL5), CXC-chemokine ligand 13 (CXCL13), CXC-chemokine ligand 5 (CXCL5) CC-chemokine ligand 20 (CCL20), CC-chemokine ligand 11 (CCL11), CXC-chemokine ligand 1 (CXCL1), CXC-chemokine ligand 9 (CXCL9), CXC-chemokine ligand 10 (CXCL10), CC-chemokine ligand 3 (CCL3) and CC-chemokine ligand 22 (CCL22) were measured from homogenized PVAT using LEGENDPLEX Mouse inflammation panel (BioLegend 7404464) according to manufacturer instructions.

#### mRNA expression analysis

PVAT, BAT and WAT was collected and homogenized as described for protein quantification. RNA was isolated using the Direct-Zol RNA MiniPrep Plus kit (Zymo-Research, Freiburg, Germany). cDNA synthesis was performed with the QuantiTect Reverse Transcription Kit (Qiagen, Hilden, Germany). Gene expression was examined using the GoTaq Master Mix (Promega, Walldorf, Germany) or Taqman fast advanced Master Mix (Thermo Fisher Scientific) by quantitative real-time PCR (QuantStudio 1, Thermo Fisher Scientific). Primer sequences are listed in Table S4 mRNA expression of genes of interest was quantified by normalization to 18S mRNA expression by the 2-ΔΔCt method. In brief, the average Ct-value was calculated from the sample duplicates for each sample and gene. The mean Ct values of the target genes were then subtracted from the mean Ct values of the housekeeping gene 18s to obtain the ΔCt value.

The mean ΔCt value of the (WT) control group was then calculated for each target gene individually to subsequently subtract the ΔCt values of the individual samples from the mean ΔCt value of the WT group to obtain the ΔΔCt value. To display the results as a relative change of the samples to the WT average, we used the following formula: 2^- (ΔΔCt)^.

#### Immunofluorescence stainings

PVAT samples were cryopreserved in OCT Tissue TEK (Sakura, Torrance, USA) and 7 μm thick serial sections were prepared. Thawed sections were fixed in ice-cold acetone or 4% PFA for 10 min. For staining of 3-nitrotyrosines, sections were additionally permeabilized using 0.1% Triton X-100. Sections were blocked with 3% BSA for 1 h and incubated with the respective primary antibody (Table S3) at 4°C overnight. Non-specific binding in vWF staining was prevented by incubation with mouse Fc-blocking solution (1:200, BD Biosciences) for 1 h. Isotype controls were applied to confirm specificity of primary antibodies. Sections were washed and incubated with fluorochrome-conjugated secondary antibodies (Table S3) for 1 h in the dark, followed by DAPI staining (1:1000 in PBS, Thermo Fisher Scientific) for 15 min. Autofluorescence of elastic fibers was suppressed using the TrueVIEW autofluorescence quenching Kit (Vector Laboratories, Newark, USA). Coverslips were mounted onto glass slides with a fluorescence mounting medium (Dako, Agilent, Santa-Clara, USA) and imaged using the BZ-9000 microscope (Keyence, Osaka, Japan). Stainings were analyzed in a blinded manner and positive signals were quantified with ImageJ (Version 1.53a).

#### HE staining and lipid droplet quantification

Frozen sections were thawed and stained with Hematoxylin and Eosin (HE). After dehydrating and drying the sections, samples were mounted with Eukitt mounting medium (Merck, Sigma-Aldrich). Images were taken with the BZ-9000 microscope (Keyence) and average lipid droplet size was quantified via ImageJ.^48^

#### Oil red O staining

Lipid droplets in mature adipocytes were stained with Oil red O. In brief, cells were washed twice with PBS and fixed with 10% formalin for 30 min at RT. Thereafter, cells were washed with distilled water and stained for 30 min at RT with 0.3% filtered Oil red O solution in 60% isopropanol (Merck Sigma-Aldrich). Micrographs were obtained with the BZ-9000 microscope (Keyence) after a final wash with distilled water.

#### Carotid ultrasound

Carotid ultrasound investigation was performed with the Vevo 3100 System (VisualSonics, Toronto, Canada). A longitudinal section scan of the common carotid artery including the bifurcation was obtained with the MX 550S transducer (25–55 MHz) at a frame rate of 230–400 fps. B-mode, M-Mode, and ECG-gated kilohertz visualization recordings were performed. Data analysis was performed with the Vevo 7 Vasc, Fujifilm software (VisualSonics).

#### Organ bath experiments

Isolated aortic rings were mounted in organ baths supplied with physiological solution (Table S5), continually oxygenated, and maintained at 38,8°C. Resting tension was adjusted to 1.1 x g. After the equilibration period, aortic rings were depolarized for 20 min with 1 mL 2 M KCl to test contractile capacity. After a 20 min regeneration phase, 10 μL 10mM prostaglandin F2alpha (PGF2α, Merck Sigma-Aldrich) were added followed by a 20 min regeneration phase. The endothelial-mediated vasoconstriction was assessed by addition of acetylcholine (acetylcholine chloride, Merck Sigma-Aldrich) in log 10^−9^ to log 10^−5.5^ M concentrations. For co-incubation experiments, PVAT of adiponectin-deficient mice (*Adipoq*^−/−^), *Mpo*^−/−^ and WT mice was incubated in Dulbecco’s modified eagle’s medium at 37°C and 5% CO_2_ for 12 h. Supernatant was collected and isolated aortic rings of *Adipoq*^−/−^, *Mpo*^−/−^ and WT mice were incubated with supernatant from *Adipoq*^−/−^, *Mpo*^−/−^ and WT mice at 37°C and 5% CO_2_ for 30 min. Subsequently, organ bath experiments were performed as described above.

### Quantification and statistical analysis

Data distribution was assessed with the Shapiro Wilks Test indicating predominantly normal distribution. Statistical analyses were performed using parametric tests unless differently outlined. Differences between groups were evaluated using Student’s t test, one-way or two-way repeated measures analysis of variance (ANOVA) followed by a Tukey post-hoc correction, respectively. Correlations were tested by simple linear regression. All samples represent biological replicates. Results are expressed as mean ± SEM, for human studies as box-whisker-plot. Statistical analyses were performed using GraphPad Prism 8.4.0 (GraphPad Software, San Diego, CA, USA) or SPSS statistics 29.0.0. (IBM, Armonk, NY, USA) for human data. We assessed associations of genotype with metabolic parameter utilizing an analysis of covariance (ANCOVA), with body weight as covariate. These analyses were performed in R version 4.4.2.(R Core Team. R: A language and environment for statistical computing. In: R Foundation for Statistical Computing, editor. Vienna, Austria2020.).

## Supplemental information

Document S1. Figures S1–S19, Tables S1–S5, and Data S1–S5

Document S2. Article plus supplemental information
