# Supplementary material for: Myeloperoxidase impacts vascular function by altering perivascular adipocytes’ secretome and phenotype in obesity
Source: Cell Rep Med. 2025 Apr 18;6(5):102087. doi: 10.1016/j.xcrm.2025.102087 (PMC12147848; doi:10.1016/j.xcrm.2025.102087)
Supplement: Document S1. Figures S1–S19, Tables S1–S5, and Data S1–S5 [file mmc1.pdf]

Cell Reports Medicine, Volume *6*

Supplemental information

Myeloperoxidase impacts vascular function

by altering perivascular adipocytes' secretome

and phenotype in obesity

Alexander Hof, Max Landerer, Philipp Peitsmeyer, Ronja Herzog, Jens Alber, Maysam Ahdab, Felix Sebastian Nettersheim, Dennis Mehrkens, Simon Geißen, Simon Braumann, Henning Guthoff, Philipp von Stein, Harshal Nemade, Felix Simon Ruben Picard, Ramona Braun, Friedrich Felix Hoyer, Jens Claus Brüning, Alexander Pfeifer, Staffan Hildebrand, Holger Winkels, Stephan Baldus, Matti Adam, Jasper Schäkel, and Martin Mollenhauer

Patient characteristics	Obese patients		Lean controls	
	n (%)	mean ± SD	*n* (%)	mean ± SD
Total *n*	33 (100%)		14 (100%)	
Age		45 ± 11		60 ± 18
Female	25 (76%)		8 (57%)	
Body mass index (kg/m^2)		53 ± 10		25 ± 4
Arterial hypertension	20 (60%)		5 (36%)	
Diabetes mellitus	13 (39%)		1 (7%)	
Coronary artery disease	0 (0%)		n/a	
Smoker	20 (60%)		5 (36%)	

Table S1: Patient characteristics of bariatric patients at baseline and lean controls.

**Flow cytometry antibodies**		
**Epitope**	**Dye**	**Specification**
Ly6C	FITC	BD Biosciences, Madrid, Spain 553104
CD 115	PerCP	Thermo Fisher Scientific, Waltham, USA 46-1152-82
Ly6G	APC	BioLegend, San Diego, USA 127614
CD45	Brilliant Violet 711	BioLegend, San Diego, USA 103147
CD64	Alexa Fluor® 647	BD Biosciences, Madrid, Spain 558539
CD11b	APC/Cyanine7	BioLegend, San Diego, USA 101226
NK1.1	PE	BioLegend, San Diego, USA 108708
TER-119	PE	BioLegend, San Diego, USA 116208
CD90.2	PE	BioLegend, San Diego, USA 105308
Ly6G	PE	BioLegend, San Diego, USA 127608
CD45R	PE	BioLegend, San Diego, USA 103208
CD19	BV605	BioLegend, San Diego, USA 115539

Table S2: Fluorescent antibodies used in flow cytometry experiments.

Epitope	Species	Dilution	Specification
Anti-Adiponectin	Rabbit	1:1000 in 1% BSA/TBST	Thermo Fisher Scientific, Waltham, USA<br>PA1-054, polyclonal
Anti-UCP1	Rabbit	1:1000 in 1% BSA/TBST	Abcam, Cambridge, UK<br>Ab155117, polyclonal
Anti-sGC	Rabbit	1:1000 in 1% BSA/TBST	Cayman Chemical, Ann Arbor, USA<br>160897, polyclonal
Anti-Gapdh	Rabbit	1:7500 in 1% BSA/TBST	Cell Signaling, Danvers, USA<br>2118, monoclonal
Anti-α-Tubulin	Mouse	1:10000 in 6% milk powder	Sigma-Aldrich, Waltham, USA<br>T6199, monoclonal
Ani-Rabbit IgG,<br>Peroxidase antibody	Goat	1:10000 in 1% BSA/TBST	Sigma-Aldrich, Waltham, USA<br>A0545, polyclonal
Anti-Ly6G	Rat	1:200 in 3% BSA/PBS	Bio Legend, San Diego, USA<br>Alexa Flour 592<br>127636, monoclonal
Anti-ICAM1	Armenian hamster	1:200 in 3% BSA/PBS	Invitrogen, Waltham, USA<br>MA5405, monoclonal
Anti-vWF	Mouse	1:200 in 3% BSA/PBS	Santa Cruz, Dallas, USA<br>SC-365712, monoclonal
Anti-Nitrotyrosine	Goat	1:200 in 3% BSA/PBS	Avantor, Radnor, USA<br>BIRBORB25786-1, polyclonal
Anti-CD68	Rat	1:200 in 3% BSA/PBS	BioLegend, San Diego, USA<br>Alexa Flour 592<br>137002, monoclonal
Anti-Goat	Chicken	1:500 in 1% BSA/PBS	Thermo Fisher Scientific, Waltham, USA<br>Alexa Flour 594<br>A21468
Anti-Mouse	Chicken	1:500 in 1% BSA/PBS	Thermo Fisher Scientific, Waltham, USA<br>Alexa Flour 488<br>A21200
Anti-Armenian Hamster	Goat	1:500 in 1% BSA/PBS	Jackson ImmunoResearch, West Grove, USA<br>Cy3 labeled<br>127-165-099
Rat IgG2a,<br>Isotype control	Rat	1:200 in 3% BSA/PBS	Bio Legend, San Diego, USA<br>Alexa Flour 594 400555
Armenian hamster IgG,<br>Isotype control	Armenian hamster	1:200 in 3% BSA/PBS	Thermo Fisher Scientific, Waltham, USA<br>14-4888-81
Goat IgG,<br>Isotype control	Goat	1:200 in 3% BSA/PBS	Thermo Fisher Scientific, Waltham, USA<br>02-6202
Mouse IgG,<br>Isotype control	Mouse	1:200 in 3% BSA/PBS	Thermo Fisher Scientific, Waltham, USA<br>31903

Table S3: Antibodies used for western blot analysis and immunohistochemical stainings.

Target	Species	5´□ 3´	3´□ 5´
18s	mouse	gtaacccgttgaaccccatt	ccatccaatcggtagtagcg
CITED1	mouse	atgccaaccaggagatgaac	aggatgcaggttgaaggatg
P2RX5	mouse	gccactggagagggttcatc	gaaggcattctcctggaggc
UCP1	mouse	cctctgcactggcactacct	ctgaaactccggctgagaag
ASC1	mouse	gctctgggctctctctgcta	ggaaagacaggctgaagcac
Pparg	mouse	gacgcggaagaagagacct	tgtcagaacgtgatttctcagc
PGC1a	mouse	tgctagcggttctcacagag	agtgctaagaccgctgcatt
CIDEA	mouse	tgacattcatgggattgcag	taaccaggccagttgtgatg
Tfam	mouse	taggaaaattgcagccctgt	gctgaacgaggtctttttgg
Cox8b	mouse	gttcccaaagcccatgtct	tgctgcggagctctttttat
Dio2	mouse	gcgatggcaaagataggtga	gaatggagctgggtgtagca
Adiponectin	mouse	tggatctgacgacaccaaaa	ctctccaggagtgccatct
TNFα	mouse	agcccccagtctgtatcctt	ccactctccctttgcagaac
IL-1β	mouse	gaccttccaggatgaggaca	aggccacaggtattttgtcg
MCP-1	mouse	ctgcatctgccctaaggtct	aaaatggatccacaccttgc
AMPK	mouse	gtcactgggatggaacaggt	agcgcttagaggcatcacat
MTS1	mouse	ggagttaaaagctcgcatgg	aggtctccgttgtgcagttt
eNOS	mouse	gaccctcaccgctacaacat	ctggccttctgctcattttc
18S	human	ggggcaagatcctcactttc	ctctggcacgctcgaact
IL1-β	human	ccacagaccttccaggagaatg	gtgcagttcagtgatcgtacagg
IL6	human	ccacaatacccccaggagaa	tcaccaggcaagtctcctca
CITED1	human	aggatgccaaccaagagatg	gtttagtgggaggggtggtt
ASC1	human	tgctgggctacttttcttgg	gtgtctgacgtggatcatgg
Adiponectin	human	cctaagggagacatcggtga	gtaaagcgaatgggcatgtt
TaqMan 18S	human	Hs99999901_s1	VIC-MGB
TaqMan UCP1	human	Hs00222453_m1	FAM-MGB

Table S4: Primer sequences and Taqman probes used for qualitative PCR analysis.

Physiological organ bath solution	
NaCl	*98 mM*
KCL	*4.6 mM*
$CaCl_2$	*3.4 mM*
$MgSO_4$	*2.4 mM*
$NaHCO_3$	*24 mM*
KH_2PO_4	*1 mM*
7-D-Glukose	*11.1 mM*
Indomethacin	*10 mM*
pH	7.35

Table S5: Components of the physiological organ bath solution.

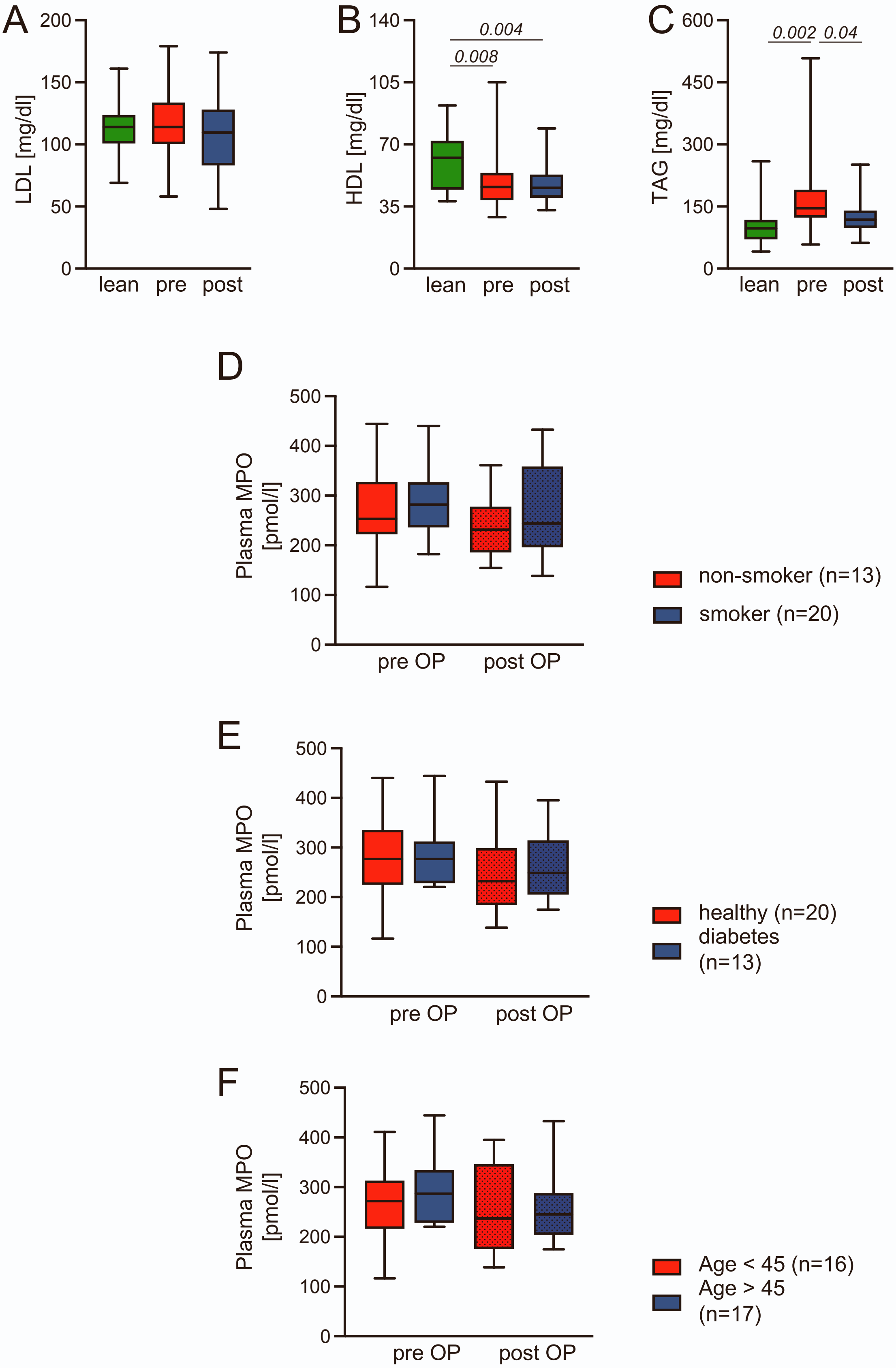

Figure S1: Plasma levels of low densitiy lipoprotein, related to Figure 1. (**A**), high densitiy lipoprotein (**B**) and triglycerides (**C**) in bariatric patients oder lean controls. MPO plasma levels relative to (**D**) smoking, (**E**) diabetes or (**F**) age of bariatric patients. For A, D-F: Statistical significance was determined by ordinary two-way ANOVA followed by Tukey's multiple comparison test. For B, C: Kruskal-Wallis followed by Dunn´s multiple comparison test was performed. n = as indicated.

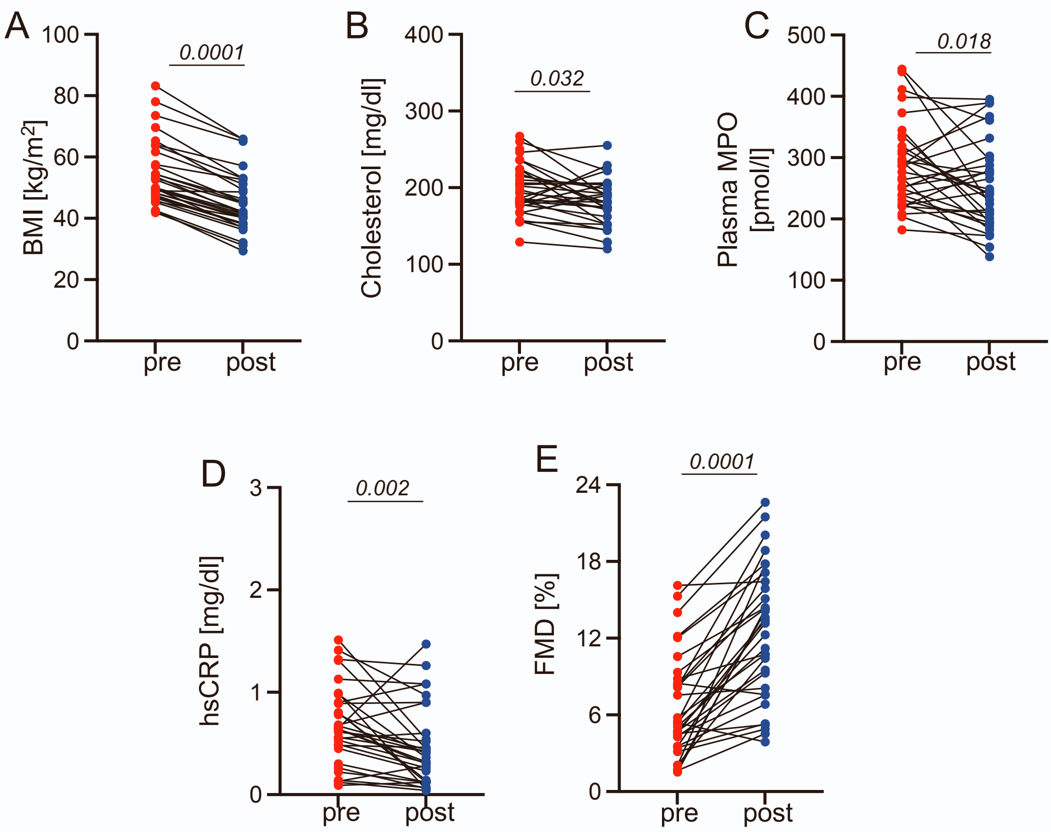

Figure S2: Bodyweight loss reduces inflammatory state and improves endothelial function in bariatric patients, related to Figure 1. Obese patients with a BMI > 37 kg/m^2 underwent bariatric surgery by Roux-en-Y gastric bypass or gastric banding. Blood samples were obtained and flow-mediated dilation (FMD) was measured before (pre) and three months (post) after bariatric surgery. BMI (**A**) and blood cholesterol levels (**B**) before and after bariatric surgery. MPO plasma levels (**C**). CRP levels before and after bariatric surgery (**D**). FMD analysis before and after bariatric surgery (**E**). *n* (patients) = 33. *p* as indicated. Statistical significance was determined by paired student's t-test.

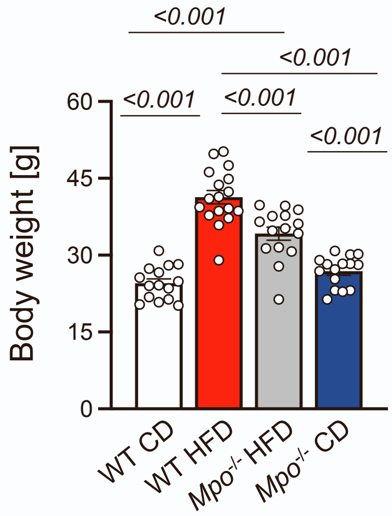

Figure S3: Body weight of WT and Mpo$^{-/-}$ mice after 12 weeks of high fat diet and respective controls, related to Figure 2. *n* = as indicated. Data is presented as mean ± SEM. Statistical significance was determined by ordinary two-way ANOVA followed by Tukey's multiple comparison test.

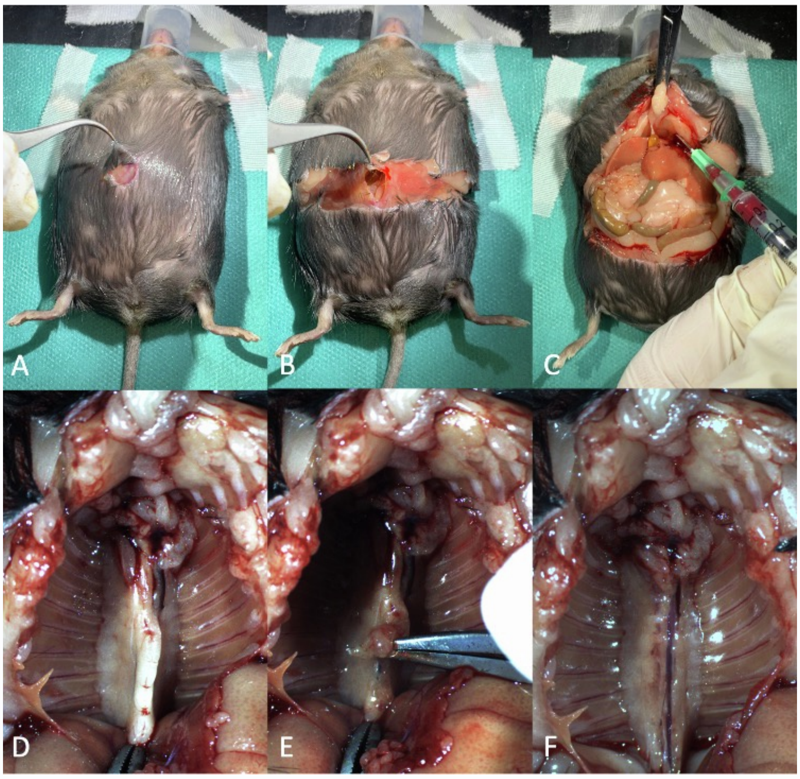

Figure S4: Sampling of blood, perivascular adipose tissue (PVAT), and the thoracic aorta, related to Figure 2. A skin incision was made under general anesthesia using isoflurane (**A**), and the peritoneum was opened (**B**). The left ventricle (**C**) was punctured through the diaphragm for blood collection. After removing lungs and heart, thoracic PVAT of the aorta became visible (**D**). The thoracic PVAT was then removed (**E**) and finally, the remaining thoracic aorta was cut out (**F**).

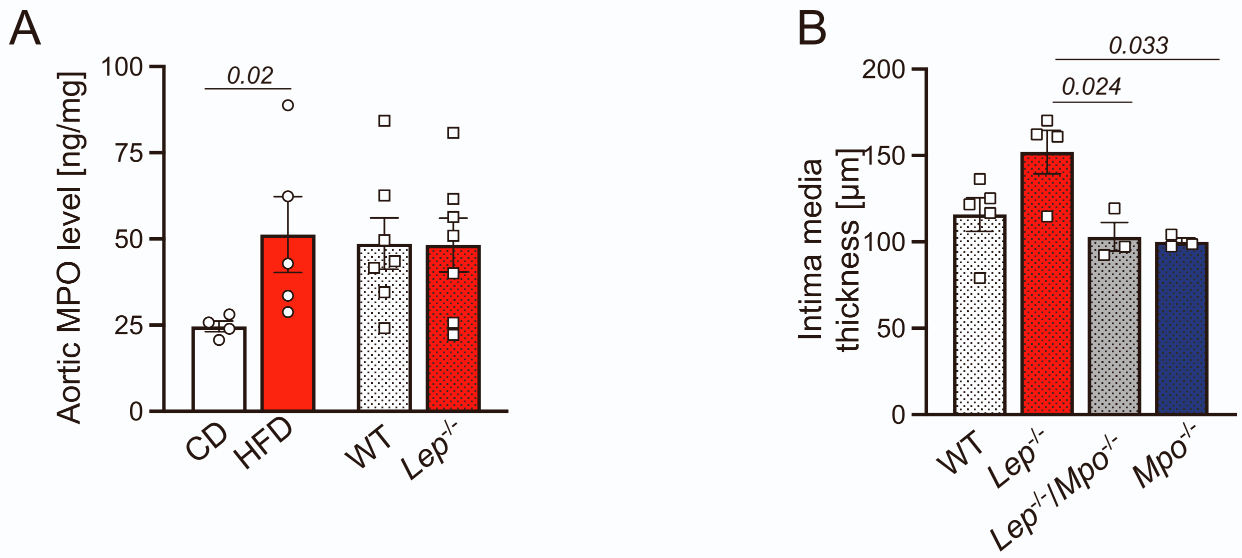

Figure S5: MPO levels in the aortic wall and intima media thickness of the carotid arteries, related to Figure 2. MPO levels in the aortic wall were significantly increased in the aortic wall of obese mice in DIO (left), whereas no alterations were observed in GIO (right). *n* = as indicated. (A) Intima media thickness (IMT) of the carotid arteries was assessed by vascular ultrasound. In Lep$^{-/-}$, but not in *Lep*$^{-/-}$/*Mpo*$^{-/-}$ mice IMT was increased compared to controls. *n* = as indicated. (B) Data is presented as mean ± SEM. For (A) statistical significance was determined by ordinary two-way ANOVA followed by Tukey's multiple comparison test. For (B) statistical significance was determined by student´s t-test.

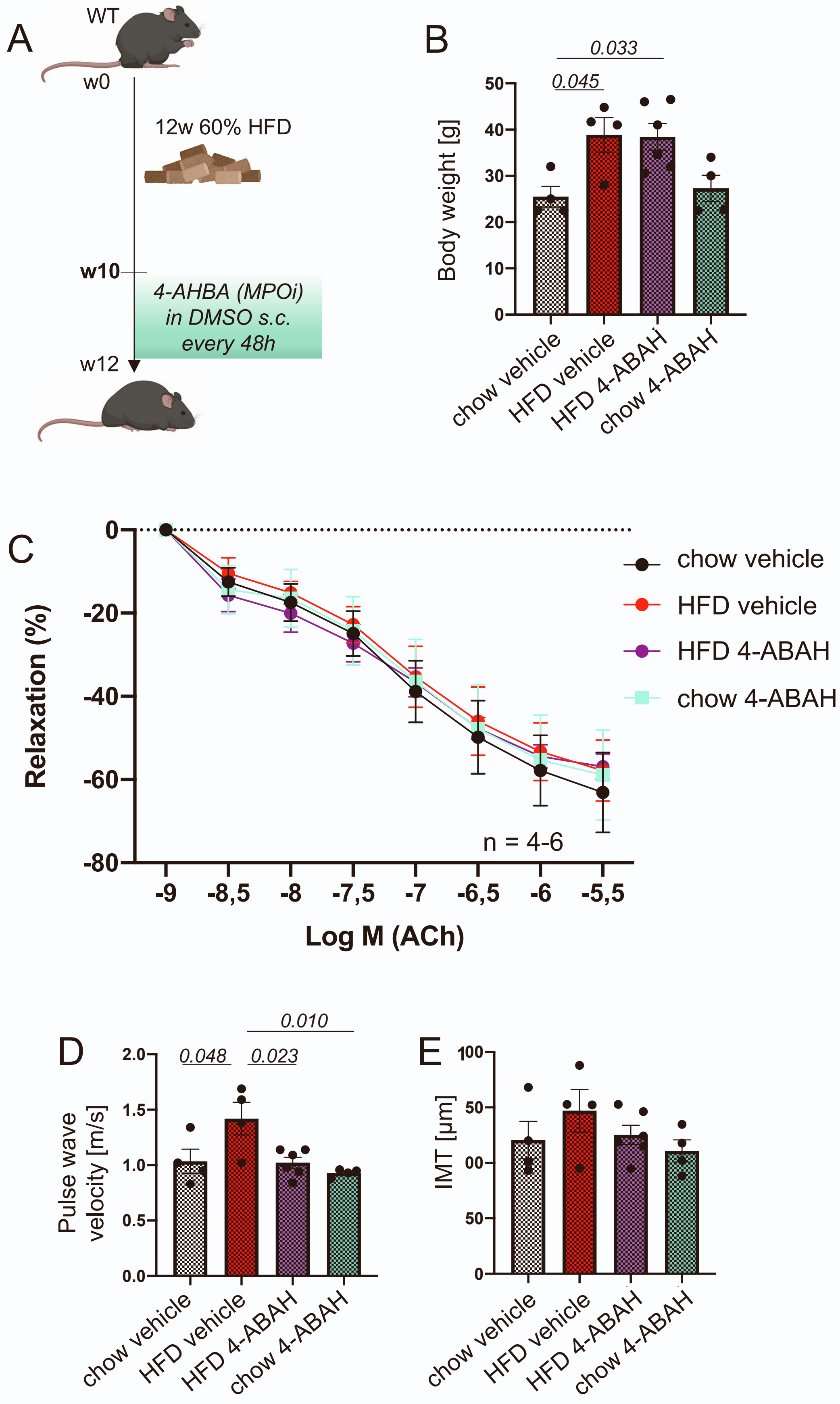

Figure S6: Pharmacological MPO inhibition in obesity, related to Figure 2. Schematic overview of the experimental set-up. Mice were treated with 20 mg/kg bodyweight 4-Aminobenzoic Acid hydrazide (4-ABAH; Nettersheim et al., Basic Res Cardiol. 2023 Sep 1;118(1):36) dissolved in 10% DMSO (14 mg/ml) s. c. or DMSO only (vehicle) (**A**). Body weight of HFD fed WT and lean controls at week 12 (**B**). Acetylcholine depended endothelium-mediated vascular relaxation of isolated aortic rings measured by organ bath investigation (**C**). Pulse wave velocity (PWV) and intima–media thickness (IMT) analyses were assessed by vascular ultrasound (**D, E**). Data is presented as mean ± SEM. *n* = as indicated. Statistical significance was determined by ordinary two-way ANOVA followed by Tukey's multiple comparison test.

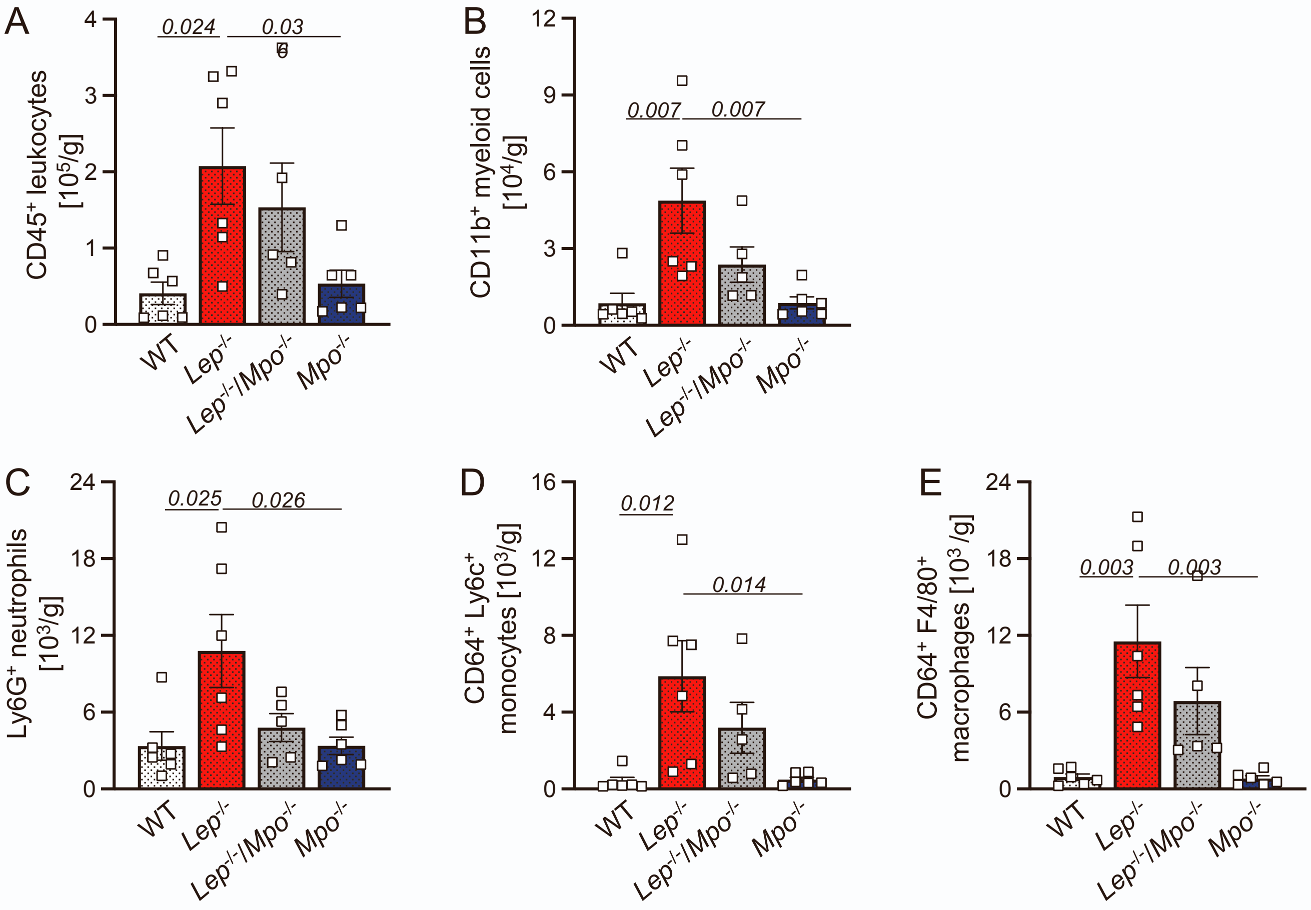

Supplementary Figure S7: MPO deficiency reduces myeloid cell frequency in perivascular adipose tissue, related to Figure 3. Leukocyte populations in GIO PVAT were analyzed by flow cytometry**.** Counts of $CD45^+$ leukocytes (**A**), CD 45^+ $CD11b^+$ myeloid cells (**B**), $CD45^+CD11b^+CD64^+Ly6G^+$ neutrophils (**C**), $CD45^+CD11b^+CD64^+Ly6c^+$ monocytes (**D**) and $CD45^+CD11b^+CD64^+F4/80^+$ macrophages (**E**) in PVAT of *Lep*$^{-/-}$ mice, *Lep*$^{-/-}$/*Mpo*$^{-/-}$ animals and lean controls. *n* = as indicated. Data is presented as mean ± SEM. Statistical significance was determined by ordinary two-way ANOVA followed by Tukey's multiple comparison test.

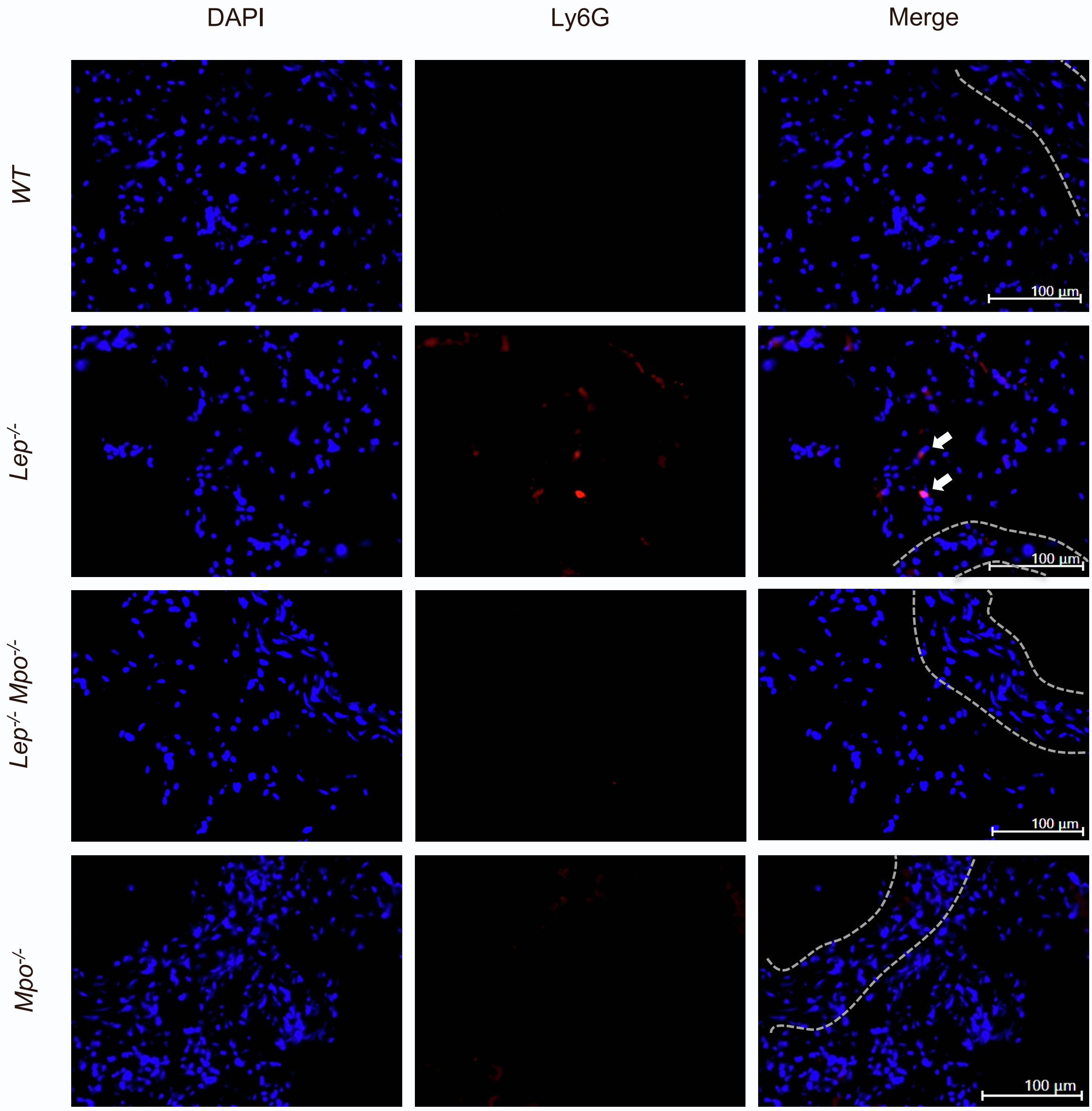

Figure S8: Immunohistochemical staining of Ly6G neutrophiles in perivascular adipose tissue, related to Figure 3. Aortic cryosections were stained for the neutrophile marker Ly6G (red, arrows) and DAPI (blue, nuclei). PVAT of *Leptin*$^{-/-}$ mice exhibited significantly more Ly6G^{+} neutrophiles per 100 cells than the *Leptin*$^{-/-}$ *Mpo*$^{-/-}$ mice and controls. Dashed lines indicate the *tunica media*. Scale bar = 100µm.

Figure S9

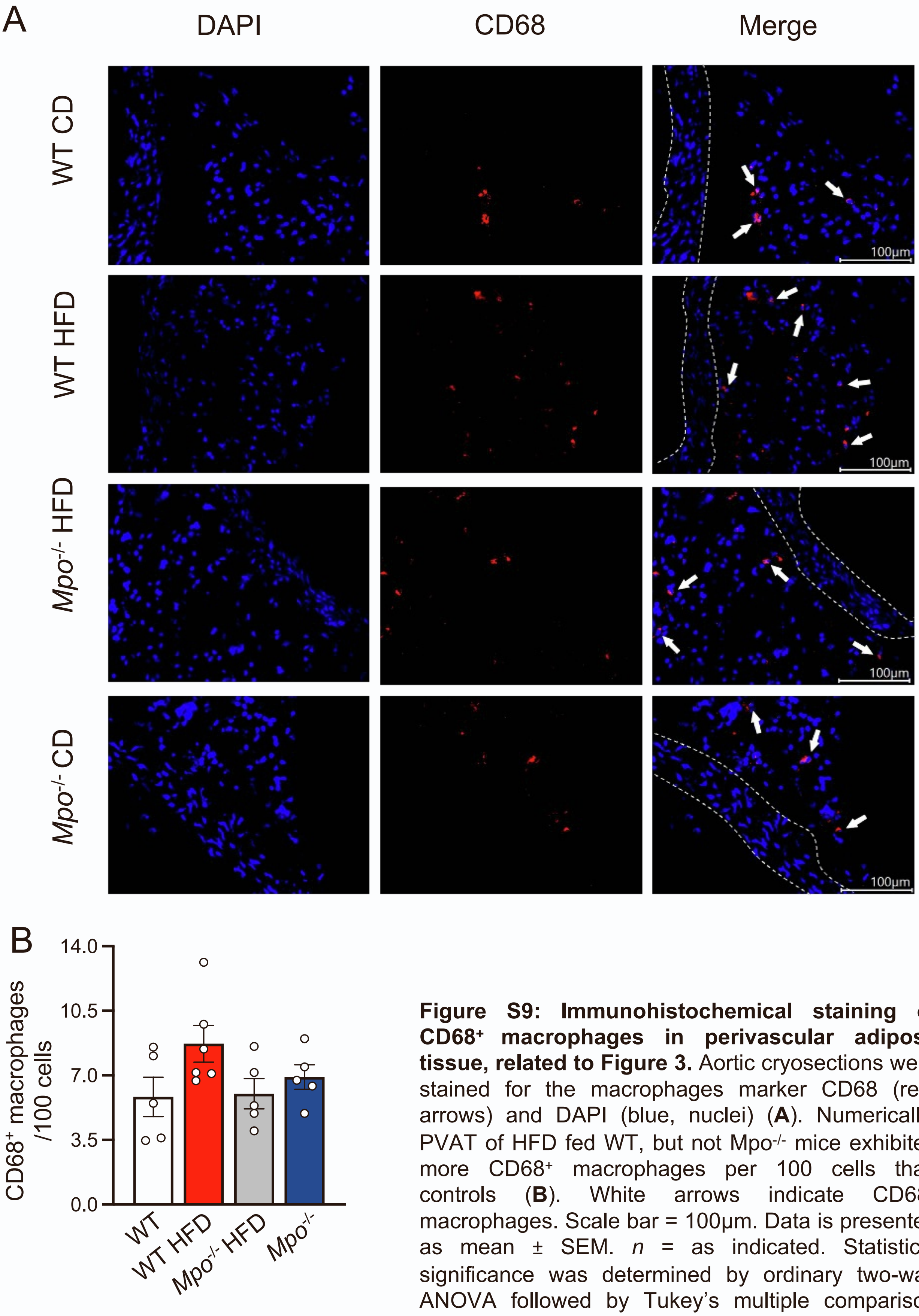

Figure S9: Immunohistochemical staining of CD68⁺ macrophages in perivascular adipose tissue, related to Figure 3. Aortic cryosections were stained for the macrophages marker CD68 (red, arrows) and DAPI (blue, nuclei) (**A**). Numerically, PVAT of HFD fed WT, but not Mpo$^{-/-}$ mice exhibited more CD68^{+} macrophages per 100 cells than controls (**B**). White arrows indicate CD68^{+} macrophages. Scale bar = 100μm. Data is presented as mean ± SEM. *n* = as indicated. Statistical significance was determined by ordinary two-way ANOVA followed by Tukey's multiple comparison test.

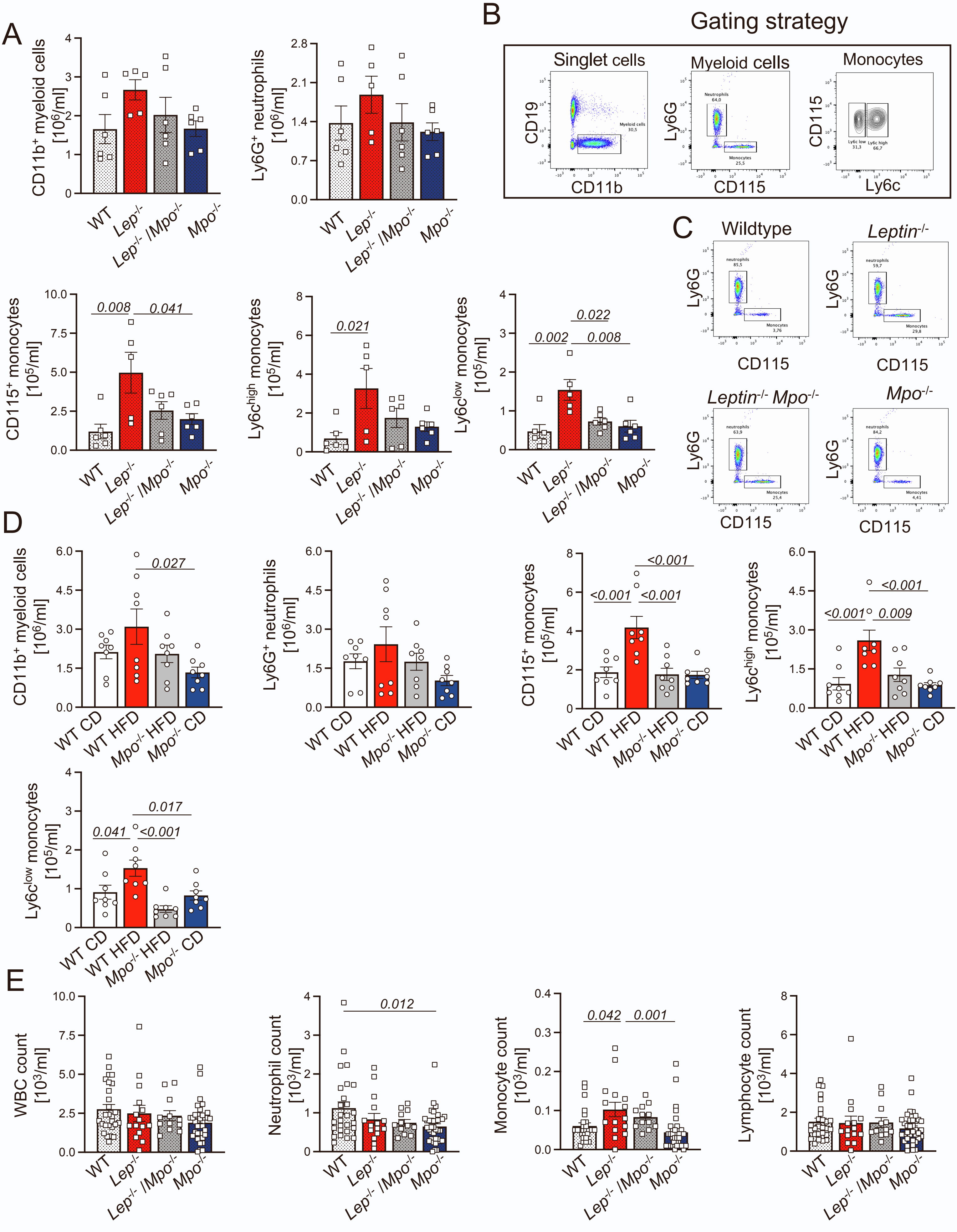

Figure S10: Quantitative changes of circulating immune cells in obesity as assessed by flow cytometry, related to Figure 3. Circulating CD11b⁺ myeloid cells, Ly6G⁺ neutrophils, CD11b⁺CD115⁺ monocytes (Ly6c^{high} and Ly6c^{low} fraction) in GIO (**A**) and DIO mice (**D**). Gating strategy is shown in (**B**) representative results of CD11b⁺CD115⁺ monocytes in GIO mice are shown in (**C**). Blood count analysis via the HemaVET Hematology System (Drew Scientific, Miami Lakes, USA) for total white blood cell- (WBC), neutrophil-, monocyte- and lymphocyte cell counts (**E**). *n* = as indicated. Data is presented as mean ± SEM. Statistical significance was determined by ordinary two-way ANOVA followed by Tukey's multiple comparison test.

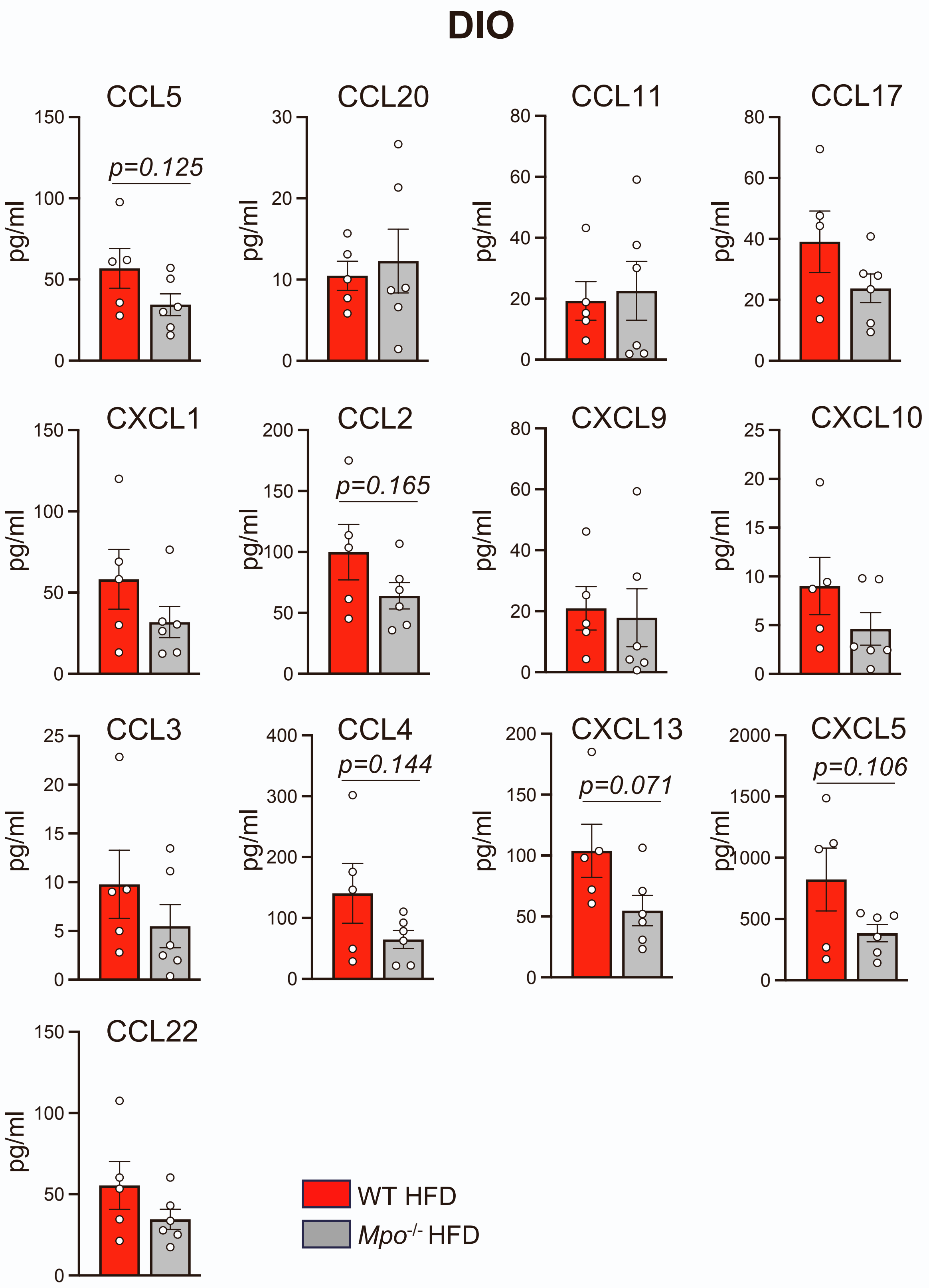

Figure S11: Proinflammatory chemokine levels in PVAT of HFD fed WT and *Mpo*$^{-/-}$ mice, related to Figure 4. Chemokine levels were determined by a multiplex bead-based assay for flow cytometry. *n* = as indicated. Data is presented as mean ± SEM. Statistical significance was determined by unpaired student´s t-test.

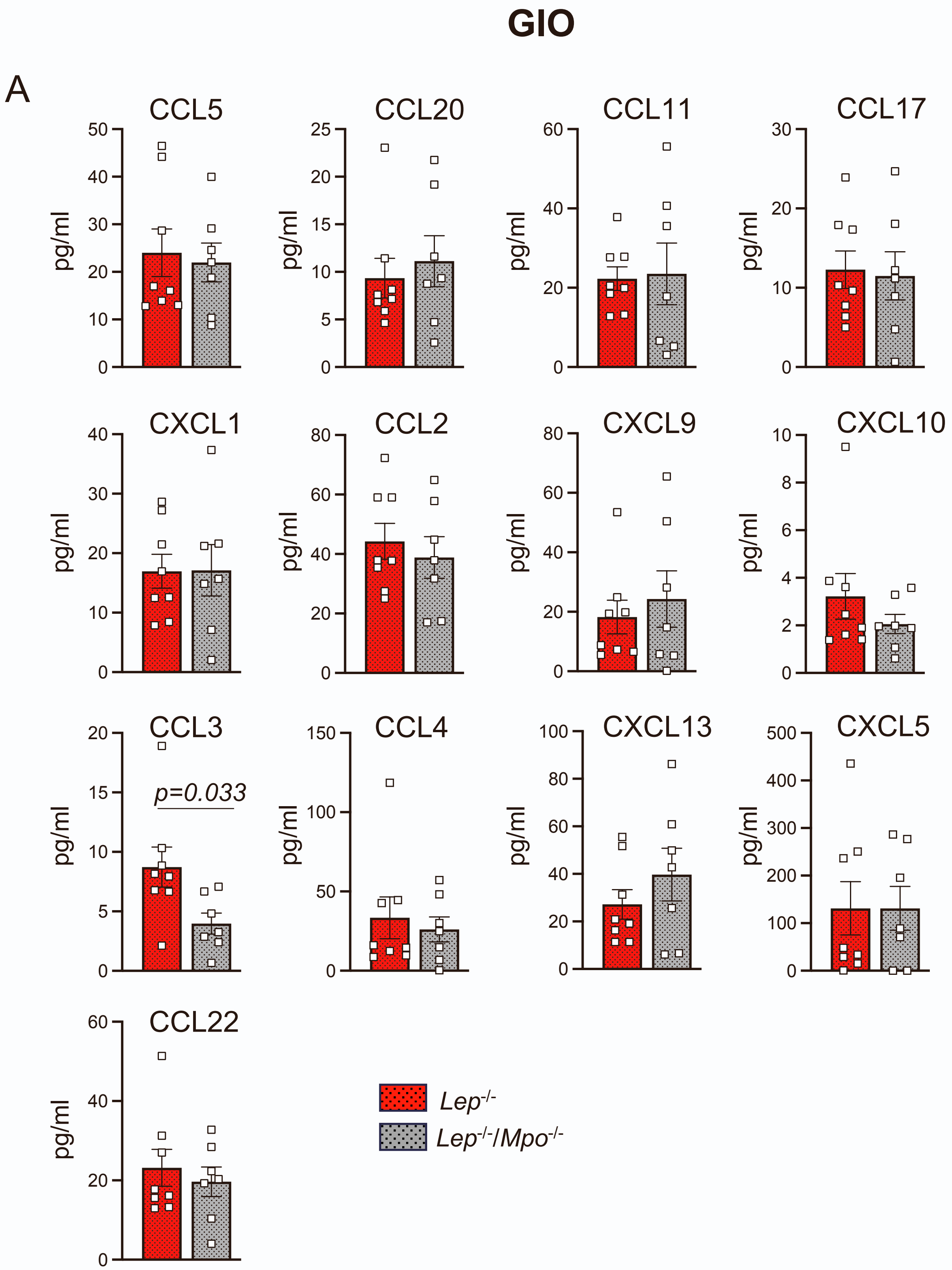

Supplementary Figure S12: Proinflammatory chemokine levels in PVAT of *Lep*$^{-/-}$ and *Lep*$^{-/-}$/*Mpo*$^{-/-}$ mice, related to Figure 4. Chemokine levels were determined by a multiplex bead-based assay for flow cytometry. *n* = as indicated. Data is presented as mean ± SEM. Statistical significance was determined by unpaired student´s t-test.

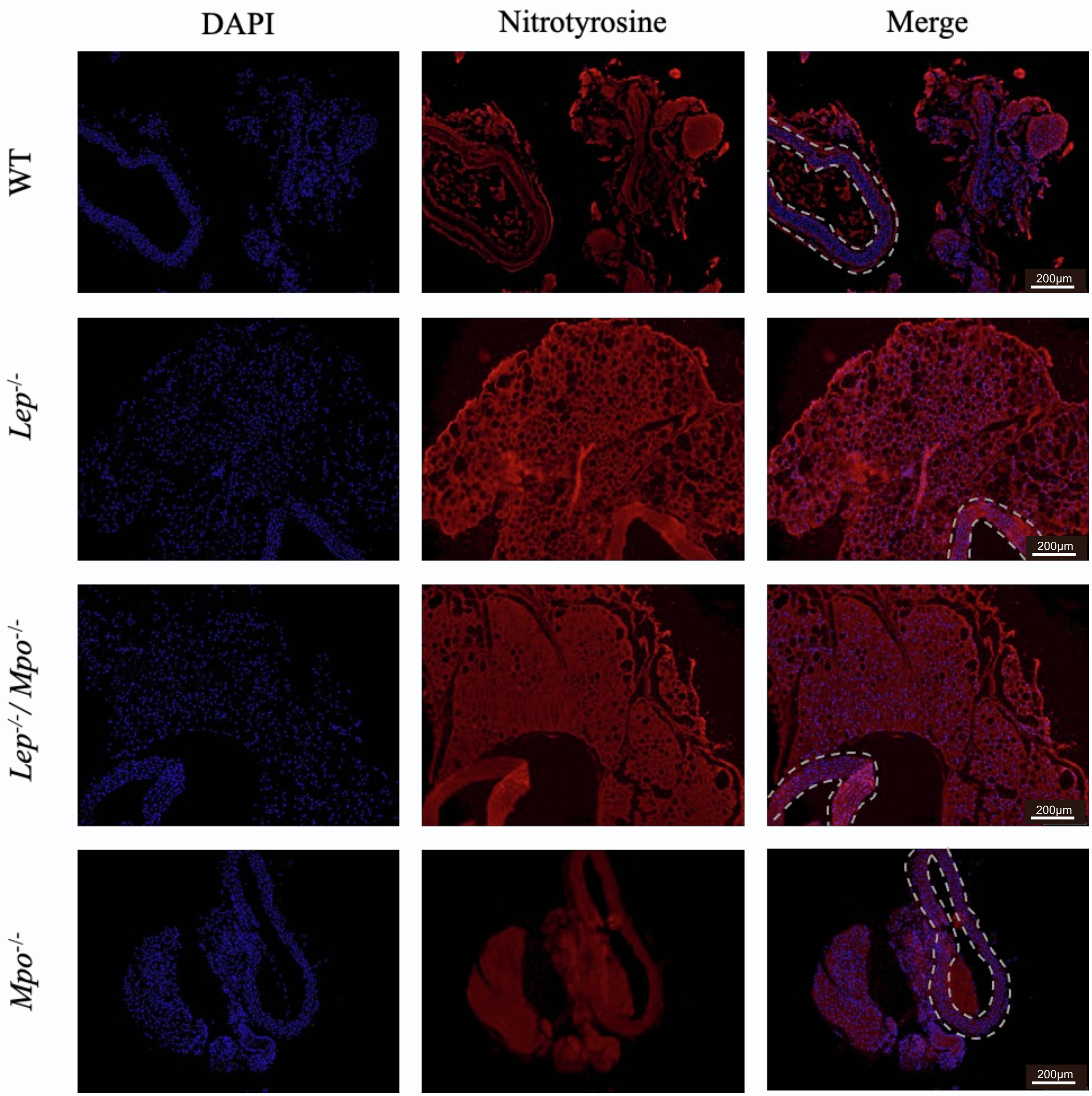

Figure S13: Immunohistochemical staining of nitrotyrosine in perivascular adipose tissue in DIO mice, related to Figure 4. Aortic paraffin sections were stained for Nitrotyrosine (red) and DAPI (blue, nuclei). Scale bar = 200µm.

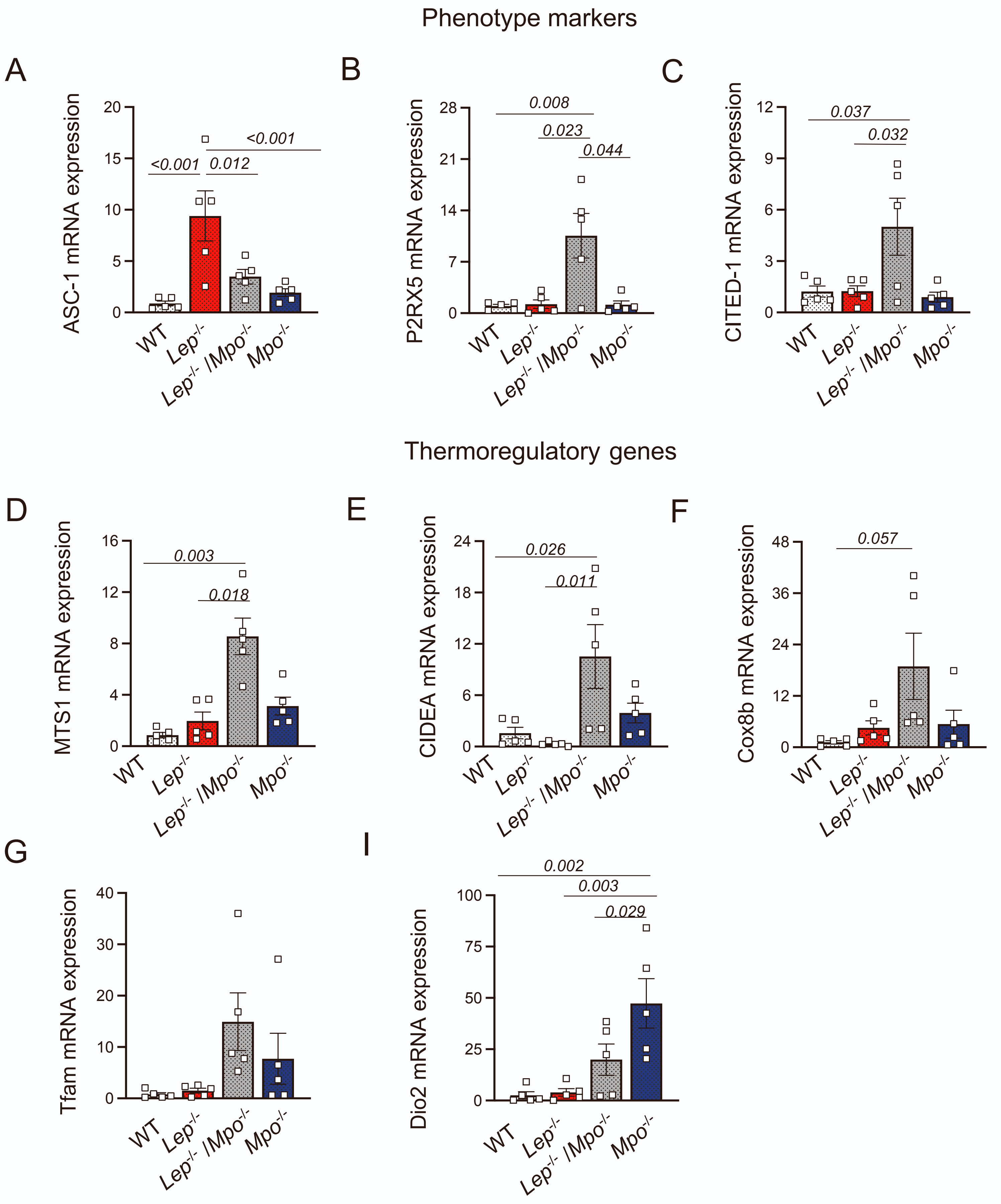

Figure S14: Myeloperoxidase impacts PVAT adipocyte phenotype and thermogenesis in obesity, related to Figure 5. The WAT marker ASC-1 was significantly higher expressed in PVAT of *Lep*$^{-/-}$ mice than in *Lep*$^{-/-}$/*Mpo*$^{-/-}$ mice (A), whereas mRNA expression of beige AT markers P2RX5 and CITED-1 was elevated in *Lep*$^{-/-}$/*Mpo*$^{-/-}$ compared to *Lep*$^{-/-}$ mice (B,C). MPO further impacts on mRNA expression of thermoregulatory genes. MTS1 and CIDEA mRNA was more abundant in PVAT of *Lep*$^{-/-}$/*Mpo*$^{-/-}$ than in *Lep*$^{-/-}$ mice (D, E), and mRNA expression of Cox8b and Tfam were significantly elevated in *Lep*$^{-/-}$/*Mpo*$^{-/-}$, but not in *Lep*$^{-/-}$ mice compared to wildtypes (F, G). Dio2 was numerically increased in *Lep*$^{-/-}$/*Mpo*$^{-/-}$, and significantly increased in *Mpo*$^{-/-}$ animals compared to *Lep*$^{-/-}$ mice (I). *n* = as indicated. Data is presented as mean ± SEM. Statistical significance was determined by ordinary two-way ANOVA followed by Tukey's multiple comparison test.

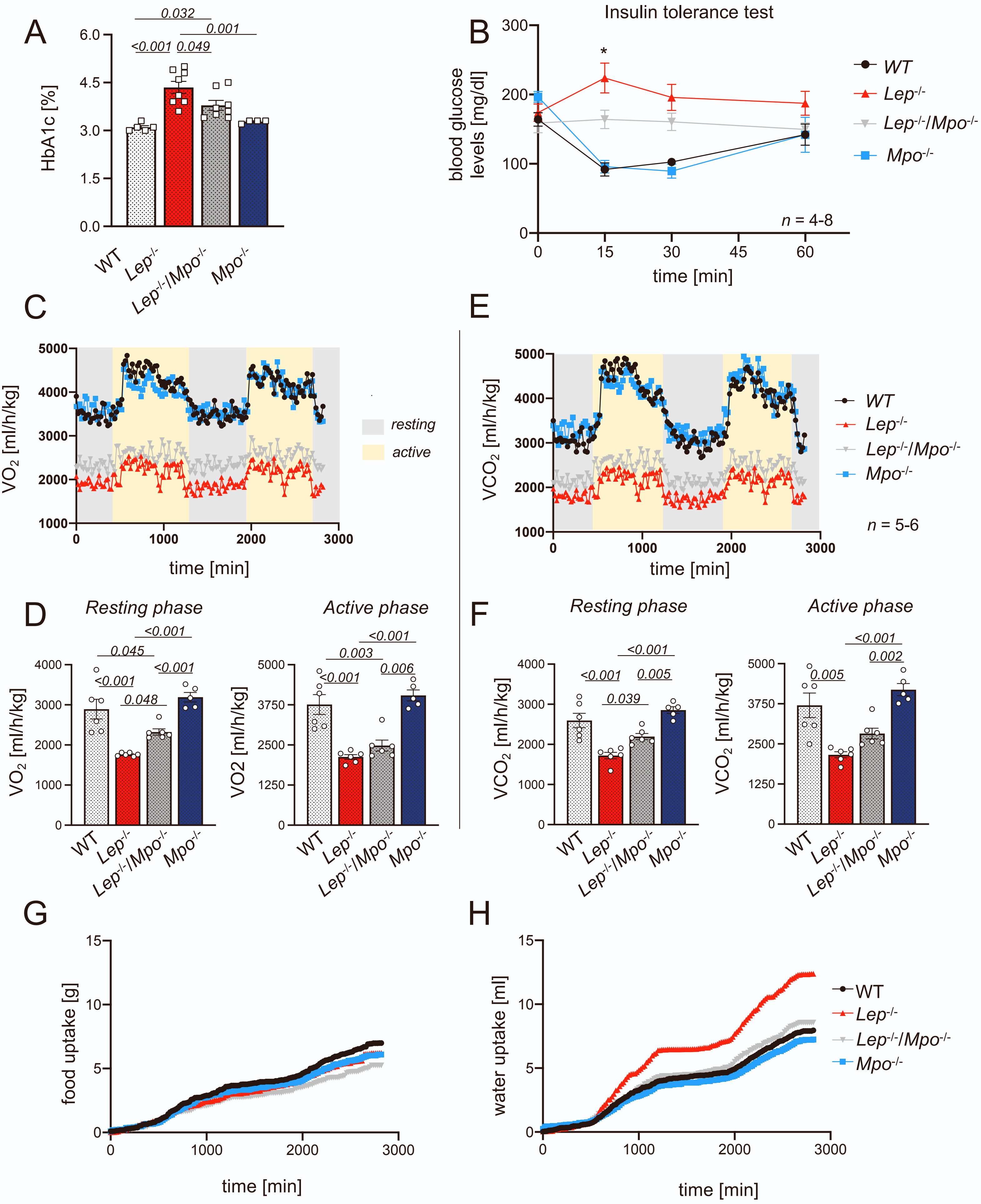

Figure S15: MPO deficiency elevates energy consumption in obesity, related to Figure 5. Plasma HbA1c levels of GIO animals (**A**). Time course of blood glucose levels after i.p. insulin injection (**B**). Oxygen consumption (**C, D**) and carbon dioxide output (**E, F**) and its respective analyses per kg BW mouse. Food (**G**) and water (**H**) uptake of GIO mice during metabolic cage analyses. Data is presented as mean ± SEM. n = as indicated. Statistical significance was determined by ordinary two-way ANOVA followed by Tukey's multiple comparison test. For (**B**): * (*Lep*$^{-/-}$ vs. *Lep*$^{-/-}$/*Mpo*$^{-/-}$) = 0.5.

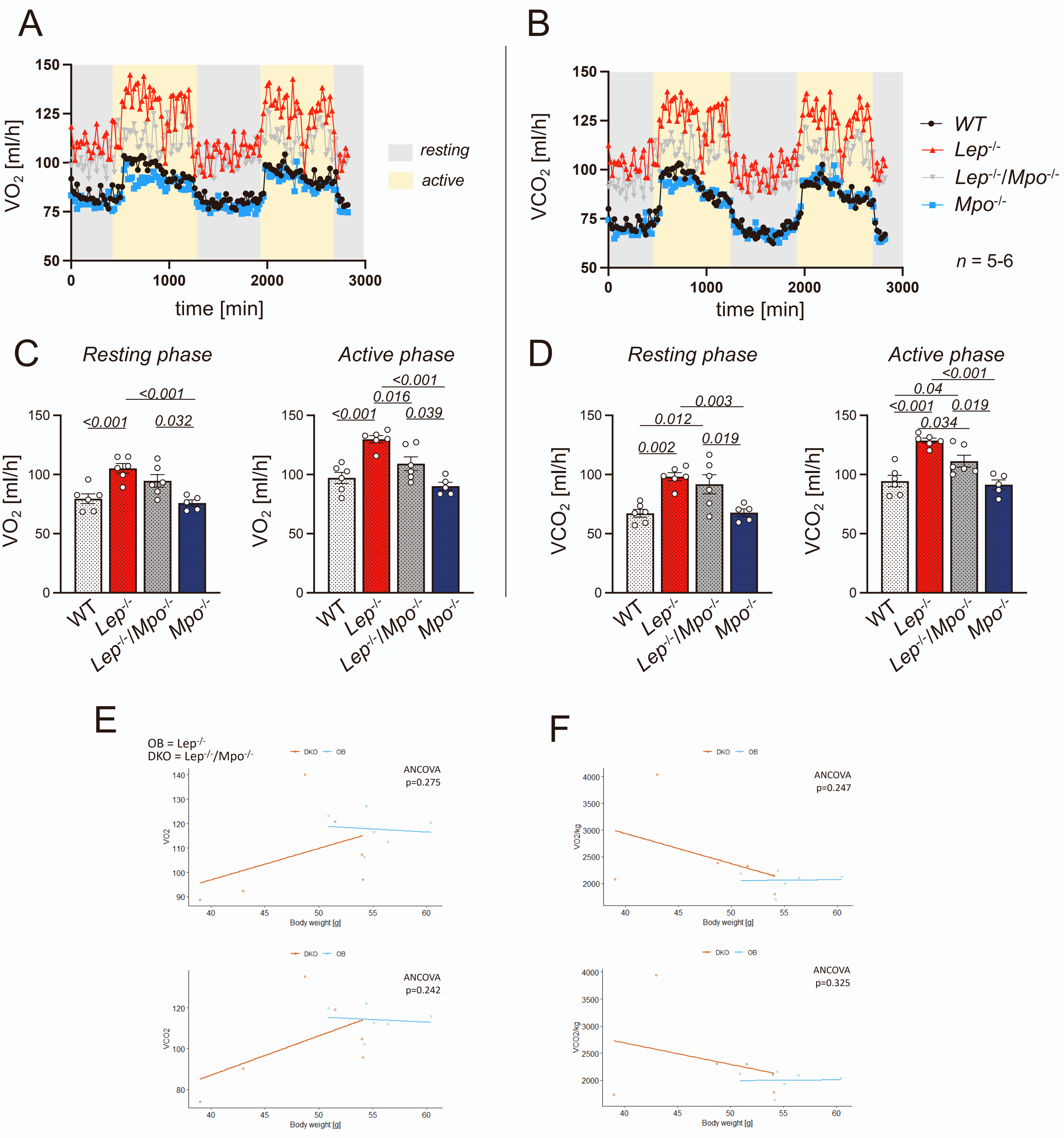

Figure S16: MPO deficiency elevates energy consumption in obesity, related to Figure 5. Oxygen consumption (**A, C**) and carbon dioxide output (**B, D**) and its respective analyses in GIO animals. ANCOVA analyses with the metabolic parameter (VO_2 and VCO_2) per mouse (**E**) and per kg (**F**) as the dependent variable, genotype as a fixed variable, and body mass as a covariate. Data is presented as mean ± SEM. *n* = as indicated. Statistical significance was determined by ordinary two-way ANOVA followed by Tukey's multiple comparison test.

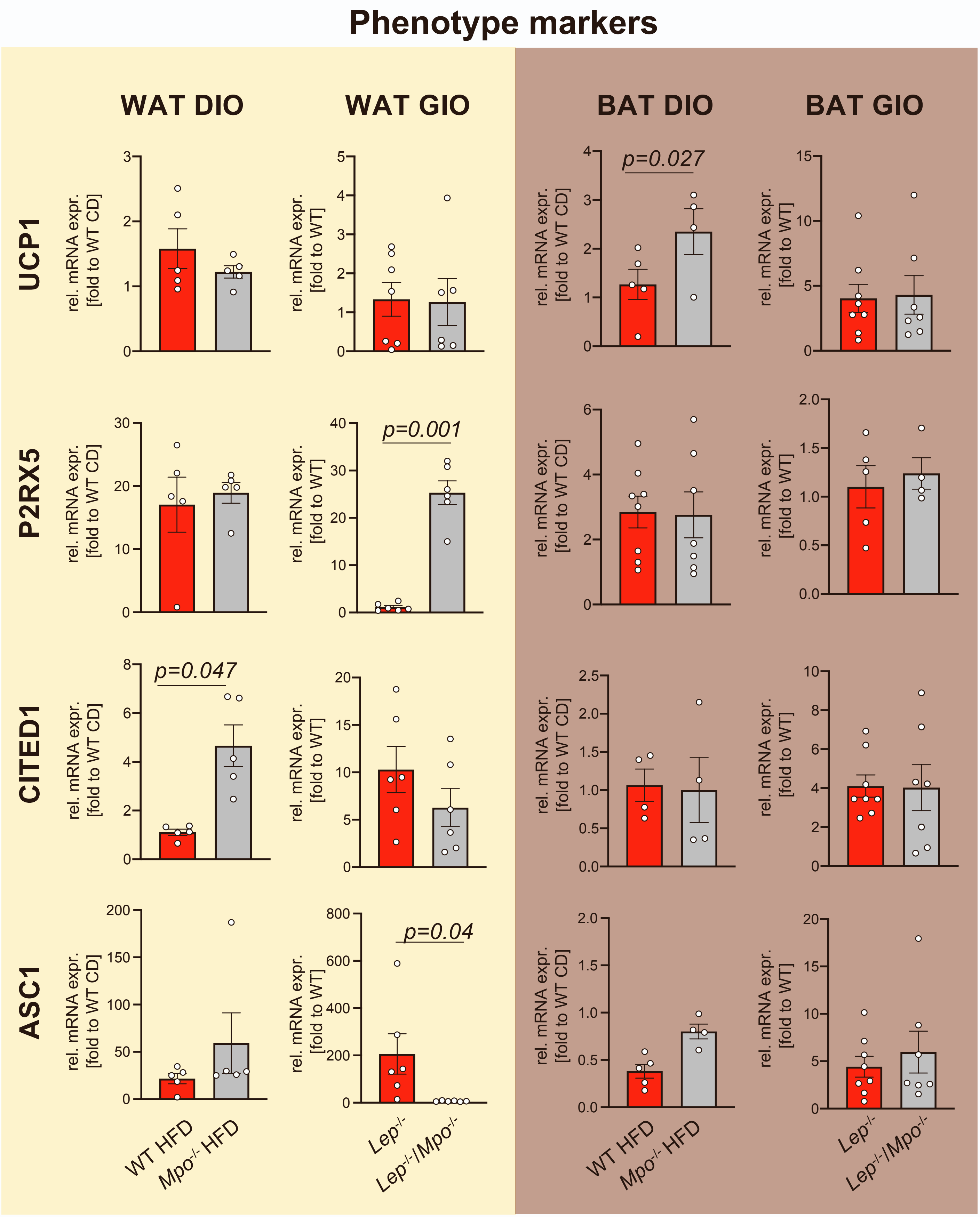

Figure S17: mRNA expression of phenotypic adipose tissue markers of WAT and BAT of DIO and GIO mice, related to Figure 5. Data is shown as relative mRNA expression fold change to control diet fed WT (DIO) or WT (GIO) respectively. Visceral white adipose tissue (WAT), interscapular brown adipose tissue (BAT), Asc-type amino acid transporter 1 (ASC-1), uncoupling protein 1 (UCP-1), Cbp/p300-interacting transactivator 1 (CITED-1). Data is presented as mean ± SEM. Statistical significance was determined by unpaired student´s t-test. n = as indicated

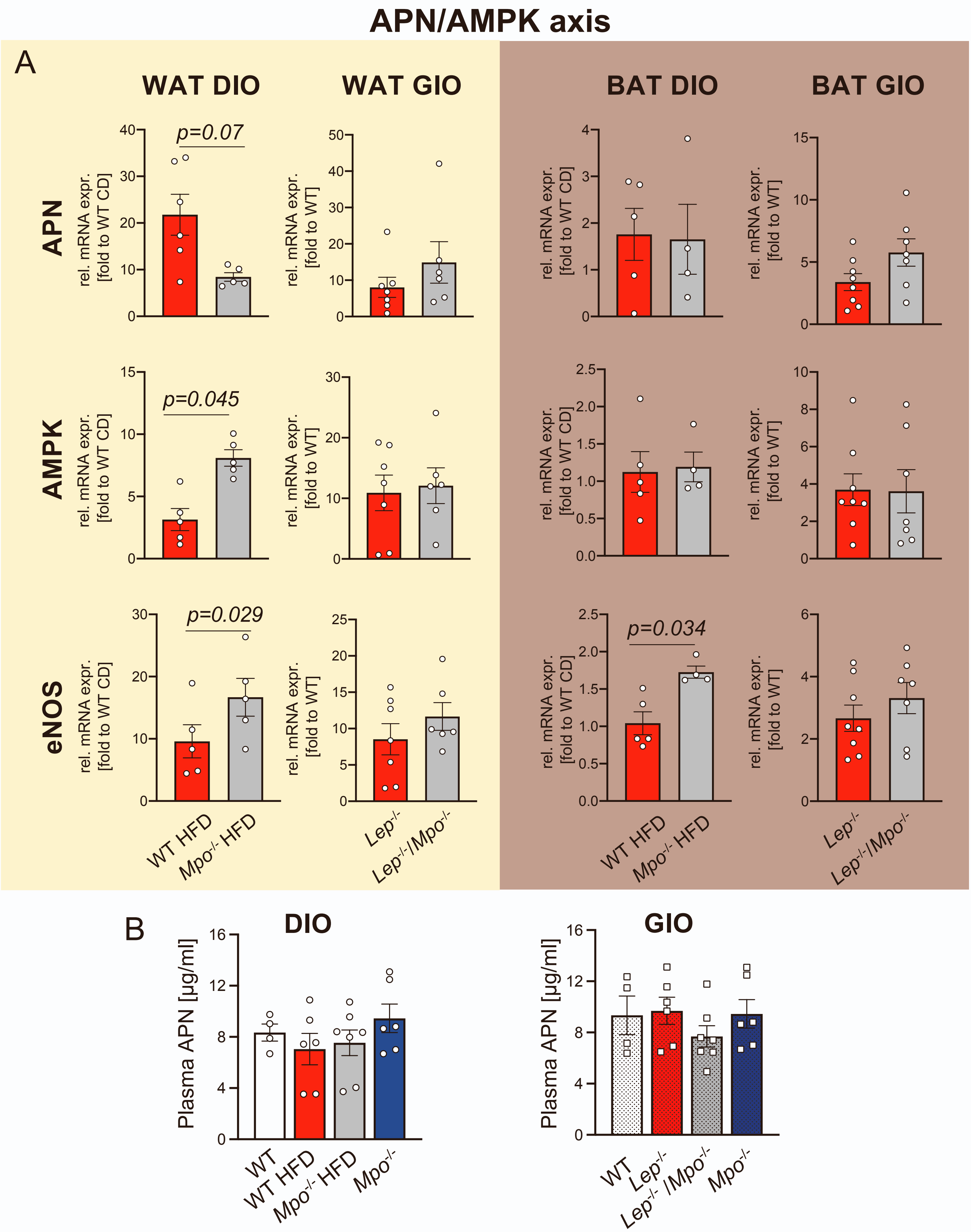

Figure S18: Systemic and non-PVAT APN levels and AMPK/eNOS expression, related to Figure 6. mRNA expression of APN and AMPK/eNOS in WAT and BAT of DIO and GIO mice **(A).** Data is shown as relative mRNA expression fold change to control diet fed WT (DIO) or WT (GIO) respectively. Adiponectin plasma level (APN) in DIO and GIO mice as indicated by ELISA (**B**). Visceral white adipose tissue (WAT), interscapular brown adipose tissue (BAT), AMP-activated protein kinase (AMPK), endothelial nitric oxide synthase (eNOS). Data is presented as mean ± SEM. Statistical significance was determined by (**A**) ordinary two-way ANOVA followed by Tukey's multiple comparison test or (**B**) unpaired student´s t-test, n = as indicated.

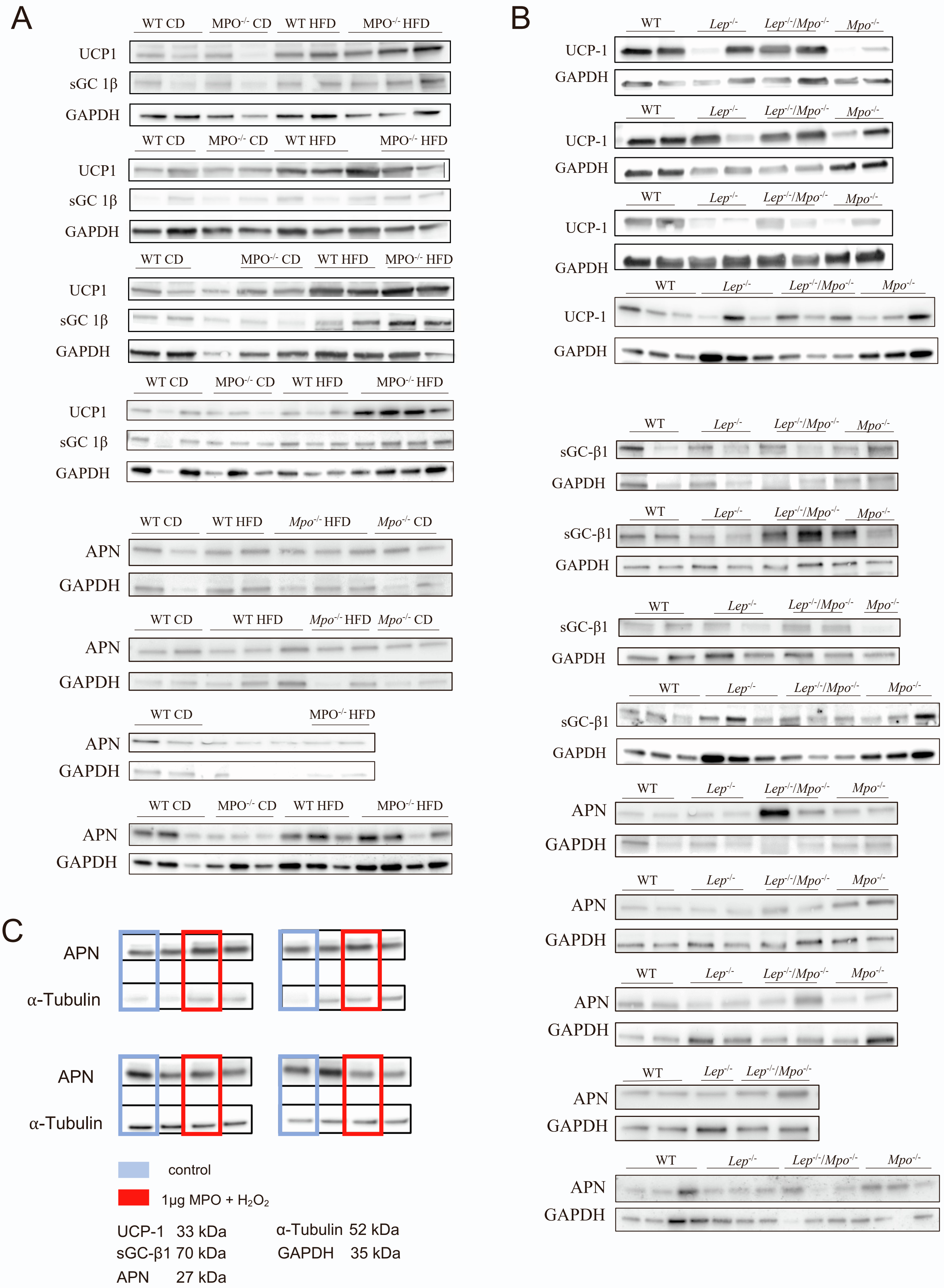

Figure S19: Blot lanes, related to Figure 5, 6 and 7. All blot lanes are shown for the dietary induced (A) and genetically inherited (B) obesity mouse model and for HWA western blots (C, blue = control; red = MPO).

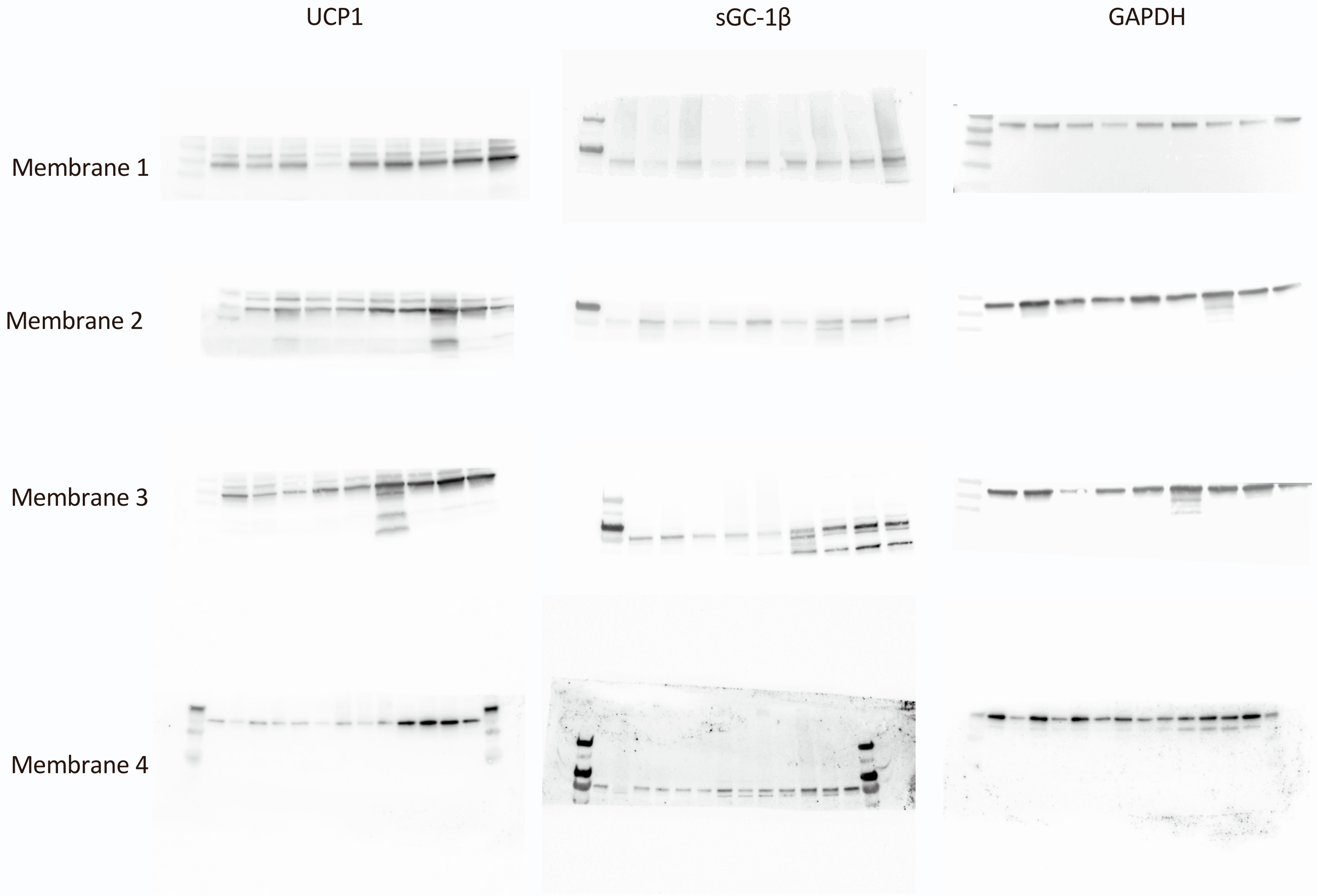

Data S1: uncropped DIO Blots (UCP1 and sGC-1β), related to Figure 5.

APN

GAPDH

Membrane 5

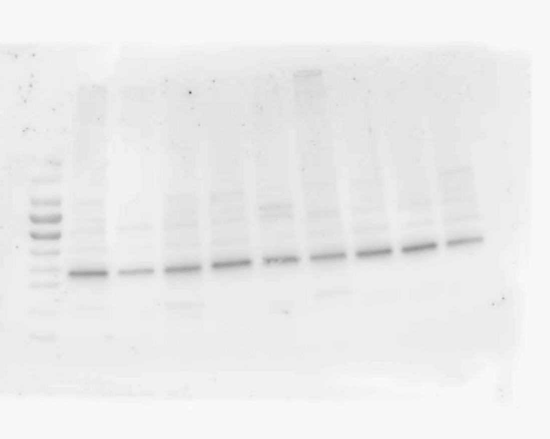

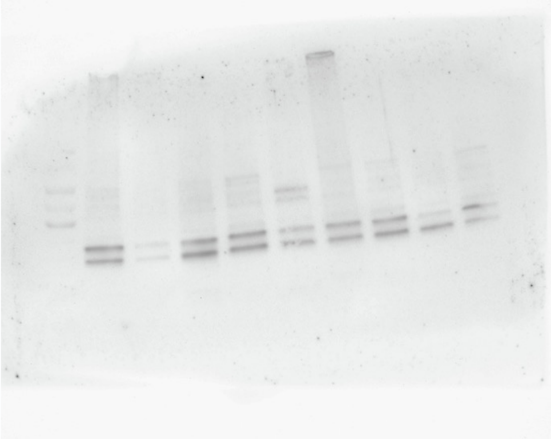

Membrane 6

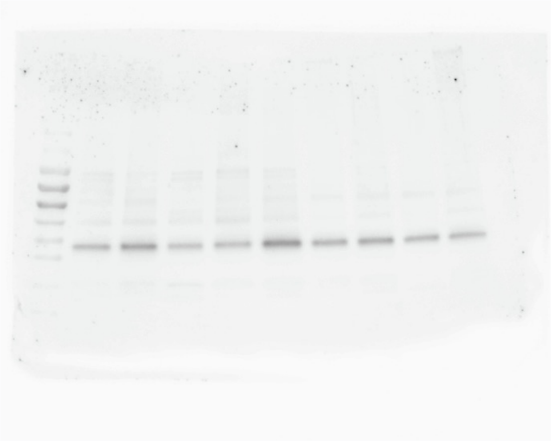

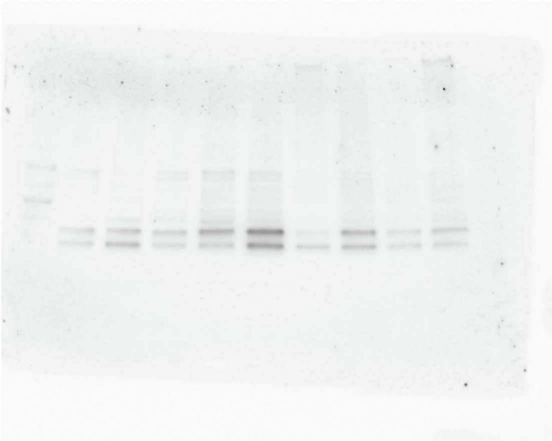

Membrane 7

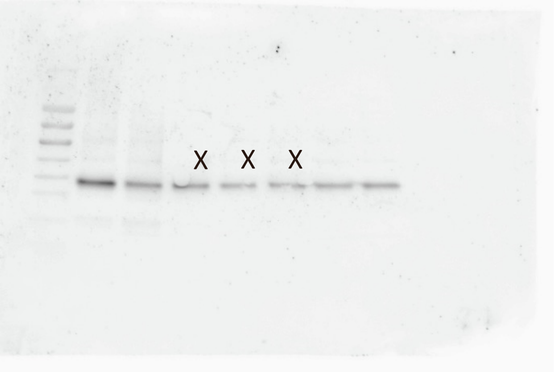

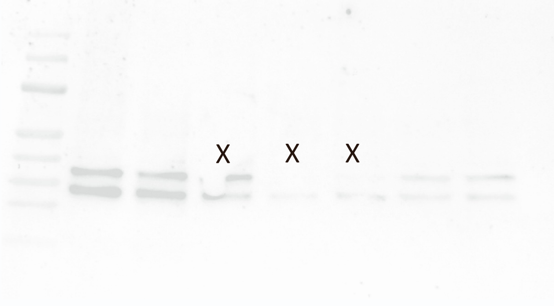

Membrane 8

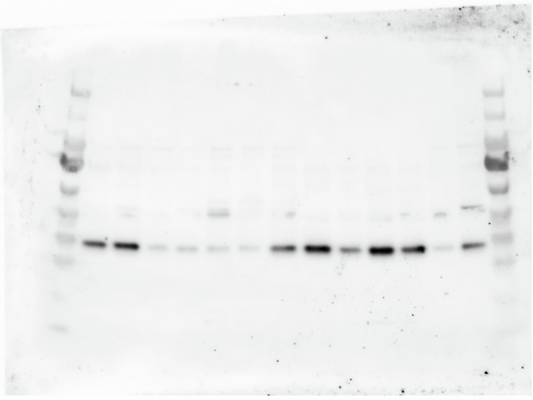

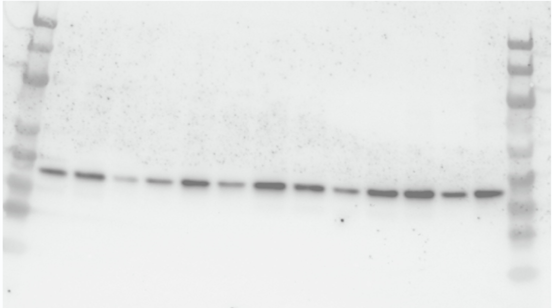

Data S2: uncopped DIO Blots (APN), related to Figure 6.

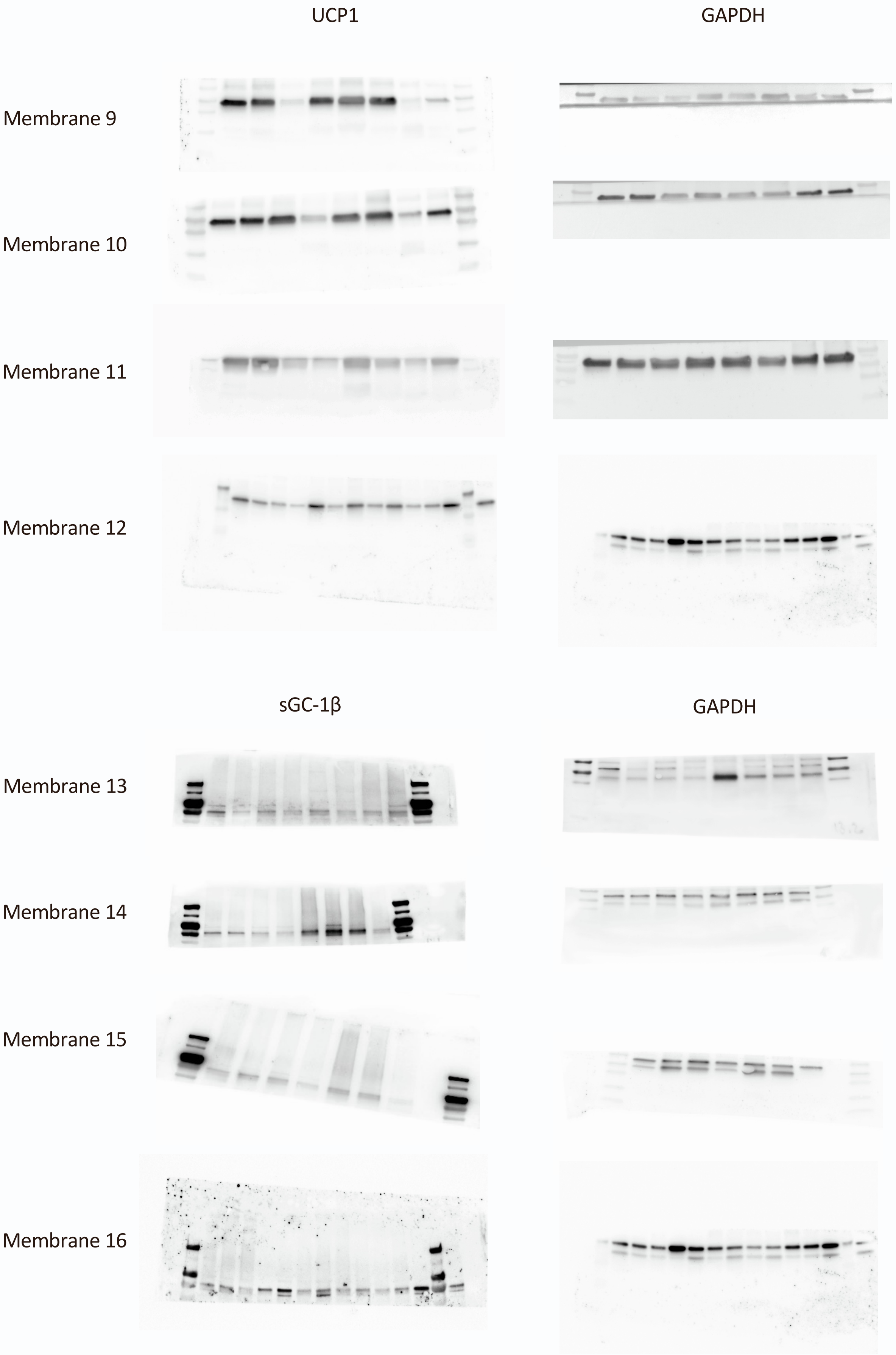

Data S3: uncropped GIO Blots (UCP1 and sGC-1β), related to Figure 5.

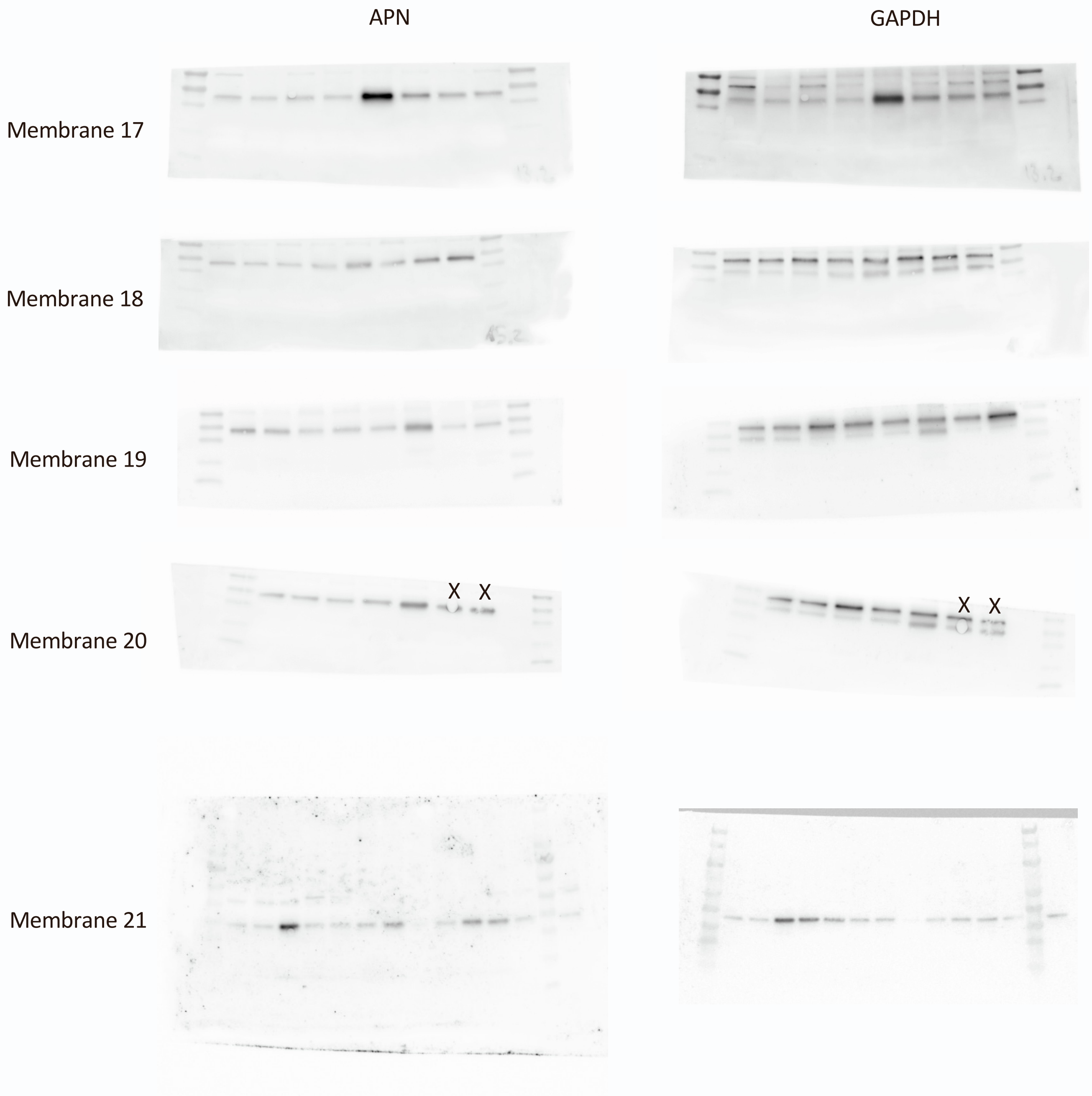

Data S4: uncropped GIO Blots (APN), related to Figure 6.

APN

alpha-Tubulin

Blot 22

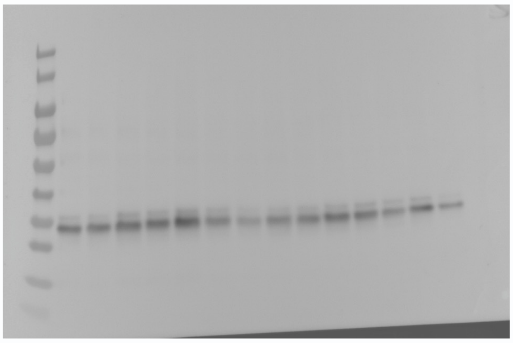

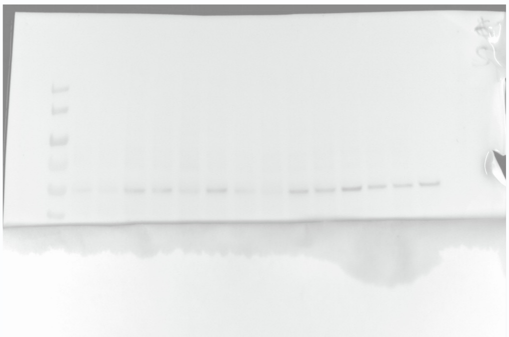

Blot 23

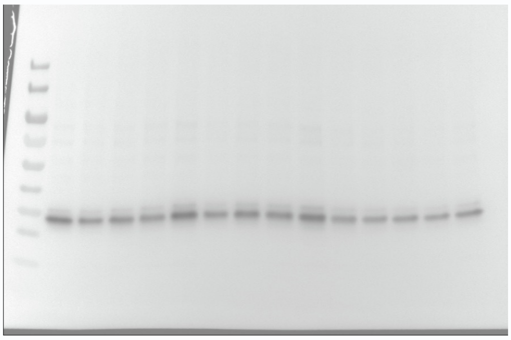

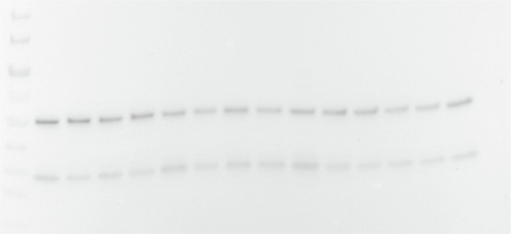

Data S5: uncropped HWP Blots, related to Figure 7.
